# QTL mapping for seedling and adult plant resistance to stripe and leaf rust in two winter wheat populations

**DOI:** 10.3389/fgene.2023.1265859

**Published:** 2023-11-23

**Authors:** Alma Kokhmetova, Nagenahalli Dharmegowda Rathan, Deepmala Sehgal, Angelina Malysheva, Madina Kumarbayeva, Makpal Nurzhuma, Ardak Bolatbekova, Gopalareddy Krishnappa, Elena Gultyaeva, Asia Kokhmetova, Zhenis Keishilov, Kanat Bakhytuly

**Affiliations:** ^1^ Laboratory of Breeding and Genetics, Institute of Plant Biology and Biotechnology (IPBB), Almaty, Kazakhstan; ^2^ Coverta Agriscience, Hyderabad, Telangana, India; ^3^ Syngenta, Jealott’s Hill International Research Centre, Bracknell, United Kingdom; ^4^ ICAR-Sugarcane Breeding Institute, Coimbatore, India; ^5^ Laboratory of Mycology and Phytopathology, All Russian Institute of Plant Protection, Pushkin, Russia

**Keywords:** wheat, QTL, yellow rust, leaf rust, adult plant resistance, all-stage resistance

## Abstract

The two recombinant inbred line (RIL) populations developed by crossing Almaly × Avocet S (206 RILs) and Almaly × Anza (162 RILs) were used to detect the novel genomic regions associated with adult plant resistance (APR) and seedling or all-stage resistance (ASR) to yellow rust (YR) and leaf rust (LR). The quantitative trait loci (QTLs) were detected through multi-year phenotypic evaluations (2018–2020) and using high-throughput DArTseq genotyping technology. RILs exhibited significant genetic variation with *p* < 0.001, and the coefficient of variation ranged from 9.79% to 47.99% for both LR and YR in all Environments and stages of evaluations. The heritability is quite high and ranged between 0.47 and 0.98. We identified nine stable QTLs for YR APR on chromosomes 1B, 2A, 2B, 3D, and 4D and four stable QTLs for LR APR on chromosomes 2B, 3B, 4A, and 5A. Furthermore, *in silico* analysis revealed that the key putative candidate genes such as *cytochrome P450*, *protein kinase-like domain superfamily*, *zinc-binding ribosomal protein*, *SANT/Myb domain*, *WRKY transcription factor*, *nucleotide sugar transporter*, and *NAC domain superfamily* were in the QTL regions and probably involved in the regulation of host response toward pathogen infection. The stable QTLs identified in this study are useful for developing rust-resistant varieties through marker-assisted selection (MAS).

## Introduction

Globally, stripe rust or yellow rust (YR) and brown rust or leaf rust (LR) are two important biotic stresses of wheat (*Triticum aestivum* L.). The YR caused by *Puccinia striiformis* (*Pst*) generally causes crop damage in the range of 0.1%–5.0%; however, crop losses can increase to 5%–25% (Wellings, 2011) based on varietal reaction and prevailing environmental conditions, and in severe conditions, crop damage can reach up to 100% (Ali et al., 2014). Most wheat-cultivating areas covering the United States, Eastern and Southern Asia, East Africa, Oceania, the Arabian Peninsula, and Western Europe are vulnerable to *Pst* incidence. The monoculture of single or closely related cultivars coupled with favorable environmental conditions is ideal for pathogen evolution. Several incidences of YR epidemics have occurred in different parts of Central Asia and Kazakhstan (Yessenbekova et al., 2016; Kokhmetova et al., 2018; Kokhmetova et al., 2021a). The YR incidence in Central and Western Asia has substantially increased between 2001 and 2010 (Morgounov et al., 2013). Recently, Central Asia recorded four YR epidemics between the years 2009 and 2014 (Ziyaev et al., 2011; Sharma et al., 2014). Historically, YR used to be confined to cool weather conditions; however, it has slowly moved to non-conventional regions due to race evolution (Muleta et al., 2017; Godoy et al., 2018). The LR caused by *Puccinia triticina* (*Pt*) is comparatively less devastating than the other two wheat rusts; however, it causes more crop damage as the frequency of its occurrence is very high and has wide global distribution (Huerta-Espino et al., 2011). The wide adaptability of this rust makes it spread to temperate areas, resulting in approximately 70% of yield losses (Herrera-Foessel et al., 2006; Aktar-Uz-Zaman et al., 2017). North Kazakhstan has reported five leaf rust epidemics during 2001–2009, causing yield damage in the range of 10%–50%, particularly in the susceptible varieties (Kokhmetova et al., 2016; Kokhmetova et al., 2021b).

Rust resistance breeding provides a sustainable solution to protect the wheat from loss of yield and grain quality. Genetically, there are two kinds of rust resistance; one is race-specific seedling resistance or all-stage resistance (ASR) and the other is race-non-specific adult plant resistance (APR) or partial resistance (Chen, 2013). Race-specific resistance is qualitative in nature, is governed by a single gene or oligogenes and only effective against a single or few races, and follows the gene-for-gene hypothesis (Flor, 1971). They express from the seedling to adult plant stage and confer vertical resistance. Generally, race-specific seedling resistance is less durable, which can easily be overcome by race evolution (Jones and Dangl, 2006). In contrast, race-non-specific resistance genes are more durable, but when used alone, they are unable to provide high levels of resistance; however, when used in combination with other race-specific or race-non-specific genes, they provide adequate resistance (Singh et al., 2000).

At present, there are 86 YR genes that were cataloged (McIntosh et al., 2020; Zhu et al., 2023); however, only a few genes like *Yr5* and *Yr15* are effective to most of the prevailing *Pst* races across the globe (Sharma-Poudyal et al., 2013). The Yr gene diversity in commercial cultivars is very important in managing stripe rust epidemics. Additionally, non-race-specific resistance driven by a few *Yr* genes such as *Yr18*, which express at the adult plant stage, confers field resistance against the three wheat rusts, which have been widely used for several decades (Randhawa et al., 2012; Krattinger et al., 2016). However, single-gene-based resistance in varieties is not enough to protect the cultivars, particularly under high disease pressure conditions (Zhang et al., 2019). Thus, the combined use of APR genes along with one or a few ASR genes may be ideal to protect the cultivars with durable resistance (Ellis et al., 2014; Liu et al., 2018). Similarly, 83 *Lr* genes have been identified (McIntosh et al., 2020; Kolmer et al., 2023), and 15 *Lr* genes exhibited APR response, including *Lr34*, *Lr46*, *Lr67*, *Lr68*, *Lr74*, *Lr75*, *Lr77*, and *Lr78*. Among them, seven are race-specific and eight are race-non-specific (McIntosh et al., 2016). Among race-specific APR genes, *Lr12*, *Lr13*, *Lr22b*, *Lr35*, and *Lr37* are qualitative in nature and provide hypersensitive reactions only at the adult plant stage (McIntosh et al., 1995; Singh and Bowden, 2011). Previous reports in Kazakhstan revealed that several *Lr* genes became ineffective due to pathogen evolution, resulting in new virulent races. Several *Lr* genes including *Lr9*, *Lr10*, *Lr19*, *Lr34*, *Lr37*, and *Lr68* are still providing resistance to several races, whereas *Lr1* has lost its effectiveness (Koishybaev et al., 2010). Some of the APR genes like *Lr1*, *Lr10*, *Lr21*, *Lr22a*, *Lr34*, and *Lr67* have been cloned; a few cloned genes like *Lr34* and *Lr67* were found to be associated with complex loci conferring resistance to multiple biotic stresses. Few pleiotropic gene complexes like *Lr19/Sr25*, *Lr26/Yr9/Sr31/Pm8*, *Lr37/Yr17/Sr38, Lr67/Sr55/Yr46/Pm46*, and *Lr34/Yr18/Pm38/Sr57* are widely used in the breeding programs across the globe, including Kazakhstan, that are still providing sufficient resistance (Kokhmetova et al., 2016; Kokhmetova et al., 2021b).

Although several race-specific seedling genes were identified for YR and LR, the genetic dissection of rust resistance in wheat through QTL mapping is equally important in the management of wheat rust as the durability of most of the race-specific seedling genes is very less, particularly under high disease pressure conditions in regions with a wide distribution of single or similar varieties. The evolution of novel races and the breakdown of race-specific genes led wheat breeders toward identifying and utilizing the durable race-non-specific APR genes and QTLs. The recent advancements in next-generation sequencing (NGS) technologies, the development of the wheat reference genome (IWGSC, 2018), and the cost reduction of genotyping made the genetic dissection of QTL regions and candidate genes more precise and effective. Previously, various mapping populations and marker systems were used to locate QTLs for LR resistance (Kolmer, 2015; Li et al., 2016; Kthiri et al., 2019; Zhang et al., 2019; Bokore et al., 2020; Ciechanowskaa et al., 2022; Delfan et al., 2023). Similarly, several QTLs were identified for SR resistance in different genetic backgrounds (Wang et al., 2015; Zhang et al., 2019; Farzand et al., 2021; Rollar et al., 2021; Yuan et al., 2020; Cheng et al., 2022; Rauf et al., 2022; Tehseen et al., 2022). However, very few are effective in providing resistance, and many among them provide race-specific resistance and hence have limited applicability to wide area deployment.

Therefore, we designed our study to identify the genomic regions that confer ASR and APR resistance to leaf and stripe rust resistance to the races prevalent across Central Asia, particularly in Kazakhstan, using two RIL mapping populations derived from Almaly × Anza and Almaly × Avocet S with multi-environment evaluations. We also attempted to provide the putative candidate genes for the identified stable QTLs to assist further validation and gene cloning experiments.

## Materials and methods

### Plant material and field experiments

The parental genotypes used to develop RILs are contrasting for both YR and LR; Almaly was the resistant parent, whereas Anza and Avocet were the susceptible parents. The RIL populations were developed by crossing Almaly × Anza (160 RILs) and Almaly × Avocet S (206 RILs) through the single-seed descent method in southeastern Kazakhstan (Supplementary Table S1). Since Almaly is the common parent in both the crosses, hereafter Almaly × Anza will be referred to as the Anza population, whereas Almaly × Avocet S will be referred to as the Avocet population. The RILs were evaluated at the seedling and adult plant-growth stages for LR and YR pathogens. The mapping populations of both the crosses along with parental genotypes were evaluated at the Kazakh Research Institute of Agriculture and Crop Production (KazNIIZiR), Almalybak (43°13′09″N and 76°36′17″E) for 2 consecutive years during 2018–19 and 2019–20 for YR and LR APR, respectively. Additionally, the RIL population of Almaly × Anza was evaluated during 2020–21 for LR APR in a randomized complete block design (RCBD) following the two replications. Each RIL was sown in a two-row plot of 1.5 m length and row-to-row spacing of 25 cm. The susceptible check variety, Morocco, was planted at an interval of every 20 plots. The RILs were also evaluated for YR and LR ASR in a greenhouse facility at the All-Russian Institute of Plant Protection (ARIPP), St Petersburg, Russia (59°73′73″N, 30°42′47″E) during 2020. Three to five seeds of each genotype were planted in 10-cm-diameter plastic pots in a disease-free area. The RILs were inoculated after 7–10 days under greenhouse conditions with three races of *P. striiformis* and six races of *P. recondita* with different levels of virulence to *Lr* and *Yr* genes (Supplementary Table S3). All entries were arranged in an RCBD design with three replications. The complete phenotypic data file of two biparental populations is provided in Supplementary Table S1.

### Phenotyping

#### Seedling resistance in greenhouse

The *P. striiformis* races were differentiated in 2020 using a set of 12 wheat lines developed in the Avocet wheat background and on nine supplemental wheat differential lines using a method developed by Johnson et al. (1972). The determination of the type of plant reaction was carried out twice within 14–20 days after infection according to the Gassner and Straib accounting scale (Gassner and Straib, 1932). At the same time, the reactions of 0, 1, and 2 points were assigned to the resistant type R (Resistant), and those of 3 and 4 points were assigned to the susceptible type S (Susceptible). The *P. triticina* races were also differentiated during 2020 using 20 near-isogenic lines (NILs) developed in the Thatcher background, each carrying one of the LR resistant genes (Kolmer and Ordonez, 2007; Schachtel et al., 2012; Kolmer et al., 2014). The virulence of the phenotypes was determined on these 20 differential lines and encoded with 0 and 1 for avirulence and virulence, respectively (Long and Kolmer, 1989; Kolmer and Ordonez, 2007). The virulence analysis tools (Schachtel et al., 2012) was used for the nomenclature of *P. triticina* races. The type of response to leaf rust was determined twice within 14–20 days after infection, according to the scale of Mains and Jackson (1926). The reactions of 0, 1, and 2 points were assigned to the resistant type R (Resistant), and those of 3 and 4 points were assigned to the susceptible type S (Susceptible).

The seedlings of the RIL population from Almaly × Avocet S cross along with the parents were inoculated with two races of *P. striiformis*, i.e., 108E187 (Pst_1) and 110E191 (Pst_2), and two races of *P. triticina*, i.e., MLTTH and TLTTR, to determine the race-specific resistance. Similarly, the seedlings of the RIL population from Almaly × Anza cross along with parents were inoculated with two races of *P. striiformis*, i.e., 108E187 (Pst_1) and 101E191 (Pst_3), and four races of *P. triticina*, i.e., THTTQ, TCTTR, TCPTQ, and THTTR. The plants were infected with spores at a three-leaf stage, and plants were placed in a humid chamber for 24 h. The seedling infection type of the RIL was scored using the same approach as that for races differentiation. The pathotypes used in this study and their virulence reaction to rust genes are provided in Supplementary Table S3.

#### Phenotyping for adult plant resistance in the field

The field phenotyping for YR and LR APR was carried out during 2018–2019 for both the populations and also during 2020 for LR APR for the Anza population at Kazakh Research Institute of Agriculture and Crop Production (KazNIIZiR), Almalybak. Pathogen racial mixtures from the local population were used to inoculate the mapping populations. The method proposed by Roelfs et al. (1992) was followed for spore sampling, storage, and propagation. The pathogen was propagated in a greenhouse on the susceptible wheat variety, Morocco. The experimental wheat material was inoculated with a mixture of spores and talc in the ratio of 1:100 by spraying with an aqueous suspension of spores with 0.001% Tween-80 at the stem elongation stages (Z21–32). After infection, the plots were wrapped with a plastic cover for 16–18 h to create high humidity. After the manifestation of diseases on susceptible control varieties, an assessment (2–3 times) of rust resistance was carried out. Leaf and yellow rust resistance of wheat accessions was evaluated using the modified Cobb scale (Peterson et al., 1948; McIntosh et al., 1995). The scoring was based both on disease severity (proportion of the leaf area infected) and on the plant response to infection (reaction type). Plant responses were recorded as resistant (R), moderately resistant (MR), moderately susceptible (MS), and susceptible (S) reactions.

### Phenotypic analysis

The phenotypic analysis was done in multi environment trial analysis with R (META-R) version 6.0 software. The best linear unbiased predictors (BLUPs) for each year and across year were used for QTL analysis. Furthermore, genetic correlation among traits and between environments, heritability, and ANOVA was done using META-R. The details of the analysis are provided in Rathan et al. (2023). Past V 3.01 was used to generate frequency distribution graphs.

### Genotyping

The genomic DNA was extracted from the parents, and each RIL was extracted from both the populations following the modified cetyltrimethylammonium bromide (CTAB) method (Dreisigacker et al., 2012). The DArTseq technology was used for genotyping of both the RILs in the Genetic Analysis and Service for Agriculture (SAGA) lab based in Mexico (Edet et al., 2018). Briefly, the sequencing of mapping populations was carried out at 192-plexing on Illumina HiSeq2500 with 1 × 77-bp reads. Allele calls for SNPs were generated through the proprietary analytical pipeline developed by DArT P/L (Sansaloni et al., 2011). Furthermore, the genetic locations of the SNPs were identified by using a 100 K consensus map given by SAGA (Sansaloni et al. unpublished). The complete genotypic data for the two biparental populations are provided in Supplementary Table S2.

### Linkage mapping and QTL detection

The linkage maps were constructed separately for Anza and Avocet RIL populations using DArTseq SNP markers. The procedure followed for linkage map construction and QTL detection is the same for both populations. The markers were filtered, and the monomorphic markers, markers with >30% missing data, high heterozygosity percentage (>30%) and low allele frequency (<5%) were removed. The BIN functionality in IciMapping 4.2 QTL software was used to remove redundant markers. A filtered set of 1,293 and 1,127 high-quality SNPs were finally used for QTL analysis in Anza and Avocet populations, respectively. The linkage map construction and QTL mapping was done in IciMapping 4.2 QTL software (Wang et al., 2012; Li et al., 2016). The Kosambi mapping function was used to construct linkage groups, using a threshold logarithm of odds (LOD) score of 3.0 (Kosambi, 1943). Within each linkage group, the marker order was carried out with the 2-opt algorithm, and rippling was carried out by maintaining a window size of 5 cM. QTL mapping was done using complex composite interval additive functionality mapping (ICIM-ADD) (Li et al., 2008). Additive QTLs were detected using a 1.0 cM incremental scan. The LOD log confidence for QTL mapping was chosen as 3.0. Then, the QTLs were localized on the respective chromosomes. One-LOD drop from the estimated QTL position was considered the confidence interval.

### *In silico* analysis

Stable QTLs with high phenotypic variation were used for the identification of candidate genes. The genes were identified in the RefSeq v1.0 assembly from the International Wheat Genome Sequencing Consortium (IWGSC) integrated in the Ensembl Plant database (https://plants.ensembl.org/index.html) using the basic local alignment search tool (BLAST). The molecular functions of the probable candidate genes found in the overlapping regions and within the 0.1 Mb flanking regions were identified. The role of the genes in governing leaf and yellow rust resistance was validated by comparing with the published literatures.

## Results

### Genetic parameters and trait associations

**FIGURE 1:**
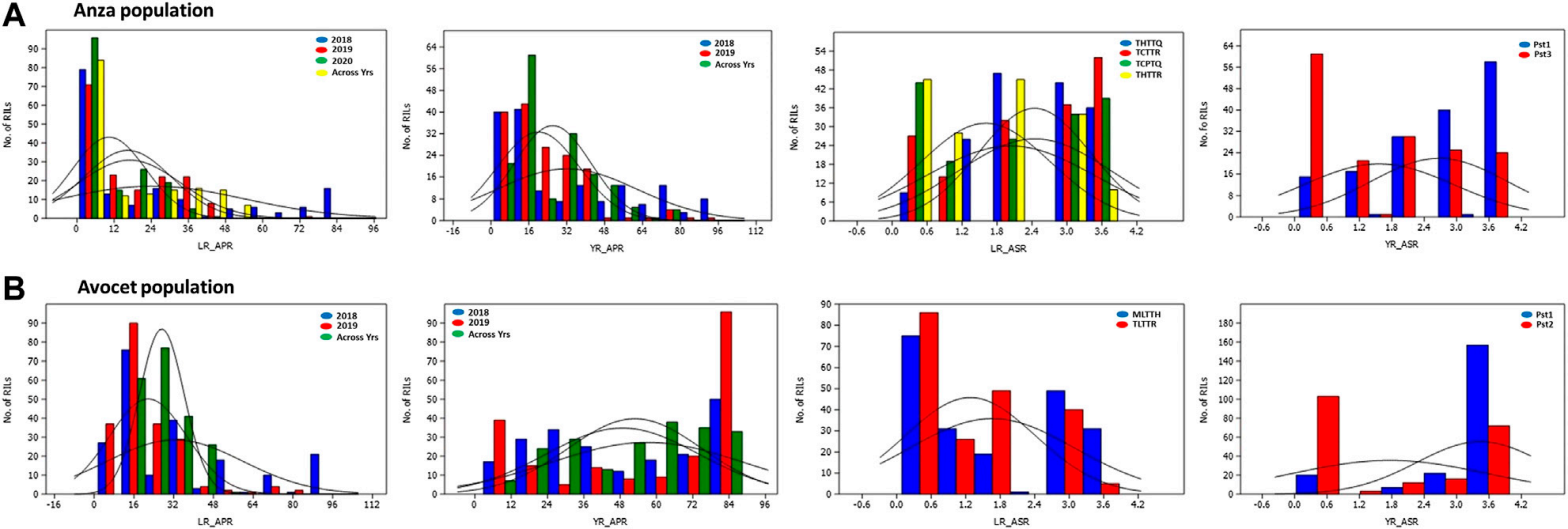
Frequency distribution graphs for LR APR, YR APR, LR ASR, and YR ASR. RILs for Anza and Avocet population. LR_ASR: leaf rust seedling resistance; YR_ASR: yellow rust seedling resistance; LR_APR: leaf rust adult plant resistance; YR_APR: yellow rust adult plant resistance; (leaf rust pathotypes at the seedling stage: THTTQ, TCTTR, TCPTQ, THTTR, MLTTH, and TLTTR evaluated in 2020; yellow rust pathotypes at the seedling stage: Pst1, Pst2, and Pst3 evaluated in 2020).

Genetic parameters of both the RIL populations derived from Anza and Avocet crosses are presented in Table 1. Wide variability exists for both YR and LR resistance in both the RIL populations for all the races, as evidenced by the presence of a highly significant genotypic variance (Table 1). The frequency distributions of YR and LR severity in the field for RILs from both populations and in the seedling stages exhibited continuous variation (Figure 1; Supplementary Figures S1, S2). The interaction between genotype and location was significant for the pooled mean of YR and LR APR for both the RIL populations. Both populations exhibited a high broad sense heritability (≥0.8) for all the traits, except the APR of yellow rust (0.71) pooled data in the Anza population and APR of leaf rust (0.47) and yellow rust (0.79) pooled data in Avocet RIL population. The CV ranged from 12.9% (YR_ASR_Pst1 in 2020) to 63.3% (LR_APR in 2020) for the Anza population. Similarly, the CV ranged from 9.79% (YR_ASR_Pst1 in 2020) to 43.49% (LR_ASR_TLTTR in 2020) for the Avocet population. Broad sense heritability estimates (h2) for leaf and yellow rust across years and different infection backgrounds were high (0.82–0.98), indicating that rust resistance can be improved by breeding (Table 1).

**TABLE 1:** Genetic parameters of Almaly × Anza and Almaly × Avocet RIL populations.

Year	Trait	Heritability	Genotype variance	Gen × Envi variance	Grand mean	LSD	CV (%)
Almaly × Anza RIL population							
2018	LR_APR	0.97	909.10***	—	24.30	15.23	32.27
	YR_APR	0.96	906.32***	—	32.04	16.49	26.58
2019	LR_APR	0.88	231.48***	—	16.54	14.71	47.99
	YR_APR	0.90	331.35***	—	20.11	15.82	41.93
2020	LR_APR	0.88	162.38***	—	10.52	12.33	63.30
	LR_ASR_THTTQ	0.82	1.12***	—	2.45	1.24	28.37
	LR_ASR_TCTTR	0.92	1.89***	—	2.46	1.11	24.01
	LR_ASR_TCPTQ	0.93	2.22***	—	2.04	1.07	27.52
	LR_ASR_THTTR	0.88	1.41***	—	1.62	1.17	38.85
	YR_ASR_Pst1	0.97	1.65***	—	2.68	0.67	12.90
	YR_ASR_Pst3	0.94	2.06***	—	1.57	0.98	32.67
Overall	LR_APR	0.87	315.88***	118.43***	17.12	18.29	43.82
	YR_APR	0.71	362.58***	256.25***	26.07	28.61	32.50
Almaly × Avocet RIL population							
2018	LR_APR	0.95	791.09***	—	32.64	17.27	27.52
	YR_APR	0.93	687.52***	—	48.67	19.64	21.25
2019	LR_APR	0.89	288.97***	—	22.01	15.49	37.77
	YR_APR	0.95	1,095.34***	—	56.96	20.49	18.71
2020	LR_ASR_MLTTH	0.94	2.21***	—	1.67	0.97	30.38
	LR_ASR_TLTTR	0.90	1.43***	—	1.29	1.05	43.49
	YR_ASR_Pst1	0.96	1.42***	—	3.43	0.65	9.79
	YR_ASR_Pst2	0.98	3.37***	—	1.77	0.71	20.56
Overall	LR_APR	0.47	177.93***	362.09***	27.33	27.09	31.67
	YR_APR	0.79	620.46***	270.97***	52.81	31.82	19.88

LSD, least significant difference, CV, coefficient of variation, LR_ASR, leaf rust seedling resistance, YR_ASR, yellow rust seedling resistance; LR_APR, leaf rust adult plant resistance; YR_APR, yellow rust adult plant resistance; LR_APR, leaf rust adult plant resistance (leaf rust pathotypes at the seedling stage, THTTQ, TCTTR, TCPTQ, THTTR, MLTTH, and TLTTR; yellow rust pathotypes at the seedling stage, Pst1, Pst2, and Pst3); *** significance at *p* < 0.001; ** significance at *p* < 0.01.

The year-wise genetic correlations between YR APR and LR APR in Anza and Avocet populations are presented in Table 2. A significant correlation was found between YR and LR APR during 2019 (*p* < 0.05) and the overall mean (*p* < 0.01) in Anza population; however, no correlation was observed in 2018. Furthermore, in the Avocet population, no correlation was observed between the traits.

**TABLE 2:** Genetic correlations of Almaly × Anza and Almaly × Avocet RIL populations.

Almaly × Anza RIL population		
Year	Trait	LR_APR
2018	YR_APR	0.13
2019	YR_APR	0.17*
Overall	YR_APR	0.43**

Almaly × Avocet RIL population		
Year	Trait	YR_APR
2018	LR_APR	0.01
2019	LR_APR	0.1
Overall	LR_APR	0.04

LR_APR, leaf rust adult plant resistance; YR_APR, yellow rust adult plant resistance; ** significance at *p* < 0.01; * significance at *p* < 0.05.

### Marker statistics

Genotyping of both Anza and Avocet populations was carried out using next-generation sequencing technology DArTseq™ (http://www.diversityarrays.com/dart-application-dartseq). A filtered set of 1,293 and 1,127 high-quality SNPs were used for linkage map construction and QTL identification in Anza and Avocet populations, respectively (Table 3). In Anza population, 539 SNPs were mapped on A subgenome, 491 SNPs on B subgenome, and only 263 SNPs on D subgenome in Anza population, whereas in Avocet cross, 482 SNPs were mapped on the B subgenome, 423 SNPs on A subgenome, and 222 SNPs on D subgenome.

**TABLE 3:** Marker distribution in Almaly × Anza and Almaly × Avocet RIL populations.

Chromosome/subgenome	1	2	3	4	5	6	7	Total
Almaly × Anza RIL population								
A subgenome	66	61	103	80	80	58	91	539
B subgenome	85	71	86	55	79	47	68	491
D subgenome	49	42	33	25	30	45	39	263
Almaly × Avocet RIL population								
A subgenome	59	59	62	78	78	9	78	423
B subgenome	91	72	73	40	74	65	67	482
D subgenome	36	33	22	21	29	36	45	222

### QTL analysis

**FIGURE 2:**
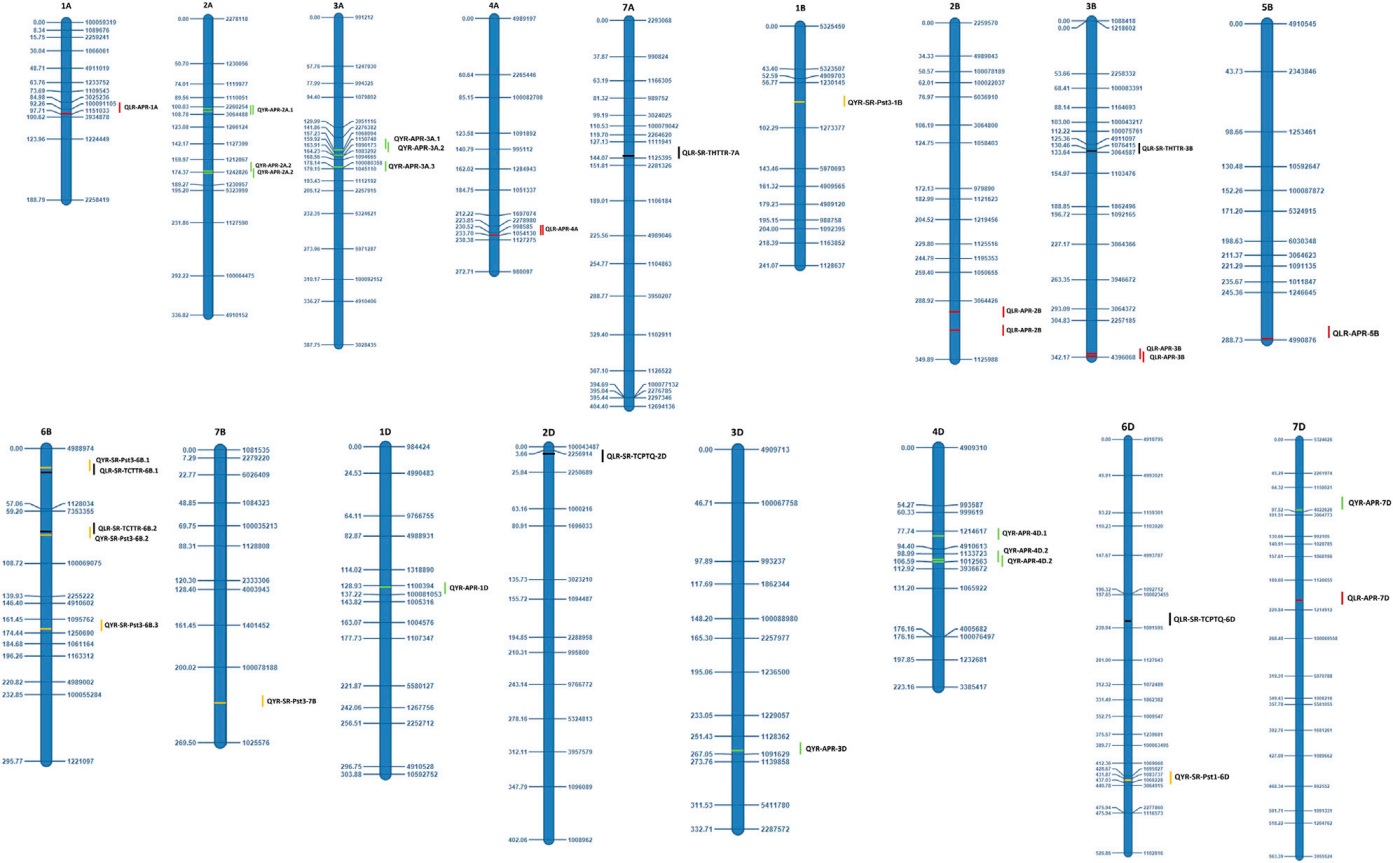
Identified QTLs and their genetic position from the Almaly × Anza population.

**FIGURE 3:**
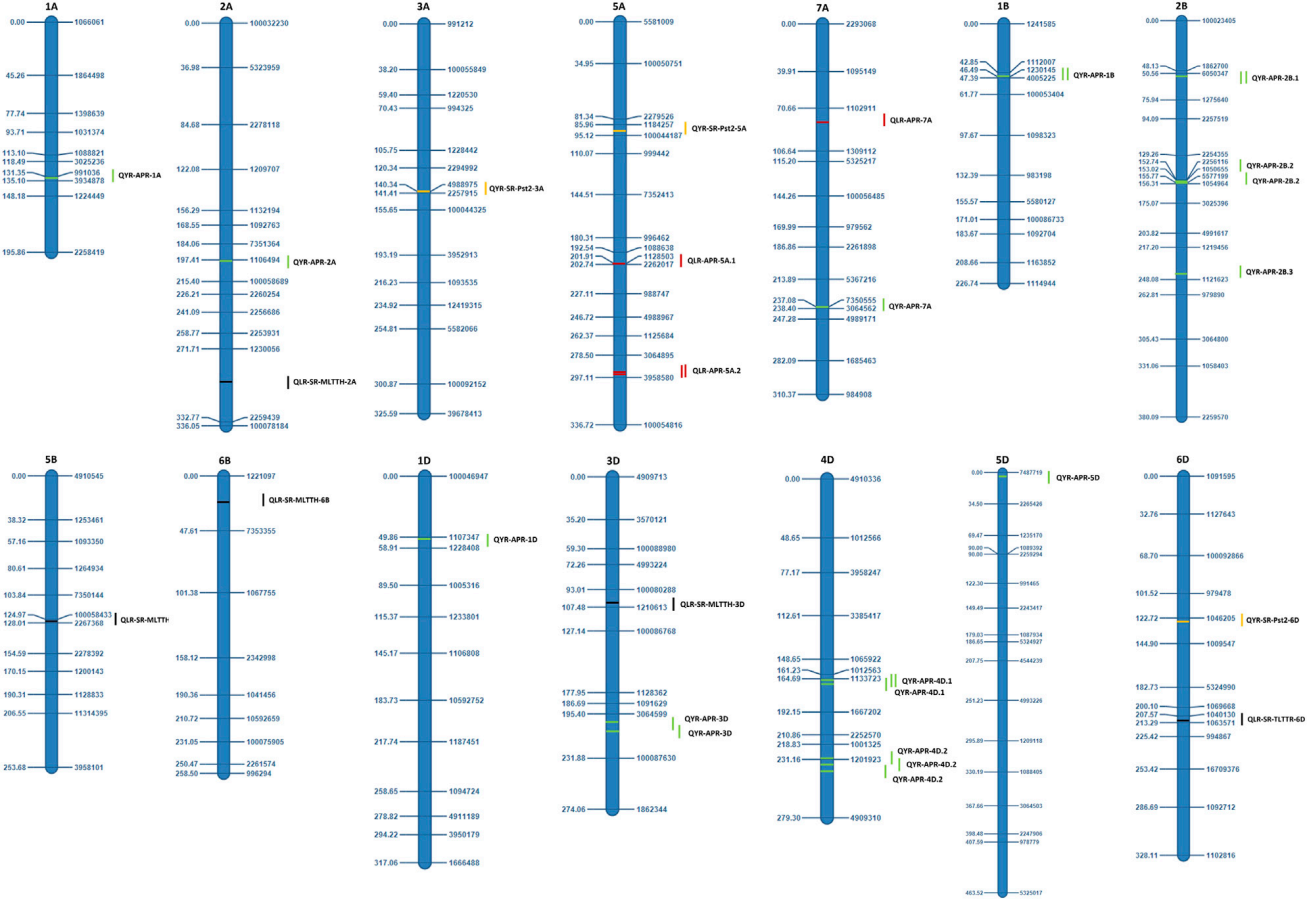
Identified QTLs and their genetic position from the Almaly × Avocet population.

The QTLs identified for APR and ASR for both the rusts are presented in Tables 4, 5 and illustrated in Figures 2, 3. A set of 51 QTLs were identified, out of which 28 QTLs included LR APR (6 QTLs), YR APR (10), and six each for LR and YR ASR in the Anza population. The remaining 23 QTLs were identified in the Avocet population including LR APR (3 QTLs), YR APR (12 QTLs), LR ASR (5 QTLs), and YR ASR (3 QTLs). Subgenome-wise 17 QTLs were identified on each subgenome of A, B, and D considering both the populations. Furthermore, the information about the favorable alleles of consistent QTLs is provided in Supplementary Table S5.

**TABLE 4:** QTLs identified for yellow and leaf rust resistance in Almaly × Anza RIL population locations for 3 years.

Year	Trait	QTL	Chr	Genetic position (cM)	Physical position (bp)	Flanking markers	LOD	PVE (%)	Add	Confidence interval
2018	LR-APR	*QLR-APR-4A*	4A	233	652,566,572–716,874,949	998,585–1,054,130	3.35	8.08	9.04	230.5–233.5
	LR-APR	*QLR-APR-5B*	5B	288	18,616,930–529,978,625	1,246,645–4,990,876	4.01	9.94	−9.72	277.5–288
2019	LR-APR	*QLR-APR-1A*	1A	97	18,885,429	100,091,105–1,151,033	3.69	6.23	4.17	94.5–98.5
	LR-APR	*QLR-APR-2B*	2B	295	785,575,596	3,064,426–1,125,988	5.83	15.18	6.39	287.5–314.5
2020	LR-APR	*QLR-APR-2B*	2B	313	785,575,596	3,064,426–1,125,988	6.23	9.41	9.36	305.5–322.5
	LR-APR	*QLR-APR-3B*	3B	342	27,980,930–457,925,422	2,257,185–4,396,068	3.03	1.16	3.19	325.5–342
	LR-APR	*QLR-APR-7D*	7D	211	42,075,096–670,266,622	1,126,655–1,214,912	3.42	4.81	6.66	200.5–222.5
Across years	LR-APR	*QLR-APR-4A*	4A	233	652,566,572 –716,874,949	998,585–1,054,130	4.34	5.76	5.39	231.5–233.5
	LR-APR	*QLR-APR-3B*	3B	340	27,980,930–457,925,422	2,257,185–4,396,068	4.16	6.37	5.51	326.5–342
2018	YR-APR	*QYR-APR-2A.1*	2A	102	683,536,393–709,771,711	2,260,254–3,064,488	6.15	9.94	10.16	96.5–106.5
	YR-APR	*QYR-APR-2A.2*	2A	174	20,557,628–34,846,399	1,212,067–1,242,826	6.86	10.39	10.40	169.5–180.5
	YR-APR	*QYR-APR-1D*	1D	129	378,730,760	1,100,394–100,081,053	3.36	4.74	−7.40	124.5–133.5
	YR-APR	*QYR-APR-4D.1*	4D	81	483,069,995–497,452,565	1,214,617–4,910,613	4.86	8.88	−9.60	77.5–87.5
	YR-APR	*QYR-APR-7D*	7D	96	637,319,694	1,158,021–4,022,626	4.37	7.34	−8.74	86.5–99.5
2019	YR-APR	*QYR-APR-3A.3*	3A	179	60,385,881	100,080,358–1,045,110	4.48	10.57	−5.31	178.5–179.5
	YR-APR	*QYR-APR-4D.2*	4D	106	495,101,244–498,684,043	1,133,723–1,012,563	3.94	9.36	−4.99	100.5–110.5
Across years	YR-APR	*QYR-APR-2A.1*	2A	106	683,536,393–709,771,711	2,260,254–3,064,488	4.95	4.07	4.65	101.5–110.5
	YR-APR	*QYR-APR-2A.2*	2A	175	9,619,406–20,557,628	1,242,826–1,230,957	6.16	4.43	4.84	169.5–182.5
	YR-APR	*QYR-APR-3A.1*	3A	159	590,301,966–603,234,500	1,068,094–1,150,748	12.67	10.35	7.65	158.5–159.5
	YR-APR	*QYR-APR-3A.2*	3A	164	567,971,052–570,530,990	1,090,173–1,083,292	23.19	20.93	−10.67	162.5–164.5
	YR-APR	*QYR-APR-3D*	3D	266	589,118,333–603,727,504	1,128,362–1,091,629	5.56	4.26	4.80	260.5–268.5
	YR-APR	*QYR-APR-4D.2*	4D	107	498,684,043–500,497,895	1,012,563–3,936,672	11.15	8.56	−6.73	104.5–110.5
2020	LR-ASR-THTTR	*QLR-ASR-THTTR-7A*	7A	144	701,561,539–713,432,629	1,111,941–1,125,395	4.31	9.07	0.37	137.5–144.5
	LR-ASR-THTTR	*QLR-ASR-THTTR-3B*	3B	131	736,747,190–754,143,752	1,076,415–3,064,587	3.14	6.71	−0.32	126.5–133.5
	LR-ASR-TCTTR	*QLR-ASR-TCTTR-6B.1*	6B	32	11,050,813–74,488,850	4,988,974–1,128,034	3.17	5.07	1.03	25.5–38.5
	LR-ASR-TCTTR	*QLR-ASR-TCTTR-6B.2*	6B	79	NA	7,353,355–100,069,075	3.53	5.14	1.05	74.5–84.5
	LR-ASR-TCPTQ	*QLR-ASR-TCPTQ-2D*	2D	4	623,163,577–641,940,538	2,256,914–2,250,689	5.02	3.70	0.52	0–9.5
	LR-ASR-TCPTQ	*QLR-ASR-TCPTQ-6D*	6D	223	485,975,428	100,023,455–1,091,595	4.15	14.63	1.05	216.5–228.5
2020	YR-ASR-Pst3	*QYR-ASR-Pst3-1B*	1B	77	630,355,967–679,858,781	1,230,145–1,273,377	5.33	4.12	1.14	71.5–82.5
	YR-ASR-Pst3	*QYR-ASR-Pst3-6B.1*	6B	28	11,050,813–74,488,850	4,988,974–1,128,034	6.47	4.24	1.16	21.5–35.5
	YR-ASR-Pst3	*QYR-ASR-Pst3-6B.2*	6B	82	NA	7,353,355–100,069,075	5.80	4.19	1.15	76.5–88.5
	YR-ASR-Pst3	*QYR-ASR-Pst3-6B.3*	6B	171	668,517,583–691,343,000	1,095,762–1,250,690	3.29	0.71	−0.47	164.5–174.5
	YR-ASR-Pst3	*QYR-ASR-Pst3-7B*	7B	234	167,570,301	100,078,188–1,025,576	7.38	4.25	−1.16	224.5–242.5
	YR-ASR-Pst1	*QYR-ASR-Pst1-6D*	6D	435	486,282,549–492,098,588	1,083,737–1,068,228	3.17	8.57	0.39	431.5–438.5

LR_ASR, leaf rust seedling resistance, YR_ASR, yellow rust seedling resistance; LR_APR, leaf rust adult plant resistance; YR_APR, yellow rust adult plant resistance (leaf rust pathotypes at the seedling stage, THTTQ, TCTTR, TCPTQ, THTTR, MLTTH, and TLTTR; yellow rust pathotypes at the seedling stage, Pst1, Pst2, and Pst3), QTL: quantitative trait locus, cM, centimorgan, LOD, logarithm of odds, PVE, phenotypic variation explained, Add, additive effect; consistent QTLs are highlighted in red font; physical position are obtained from the reference genome IWGSC, RefSeq v2.0.

**TABLE 5:** QTLs identified yellow and leaf rust resistance in Almaly × Avocet RIL population locations for 3 years.

Year	Trait	QTL	Chr	Genetic position (cM)	Physical position (bp)	Flanking markers	LOD	PVE (%)	Add	Confidence interval
2018	LR-APR	*QLR-APR-5A.1*	5A	202	84,082,776–382,970,680	1,128,503–2,262,017	6.19	2.79	9.87	201.5–202.5
	LR-APR	*QLR-APR-5A.2*	5A	294	80,081,823–607,673,215	3,064,895–3,958,580	3.86	2.17	−8.93	286.5–297.5
	LR-APR	*QLR-APR-7A*	7A	88	18,568,134–254,687,847	1,102,911–1,309,112	3.56	11.77	21.99	86.5–90.5
Across years	LR-APR	*QLR-APR-5A.2*	5A	293	80,081,823–607,673,215	3,064,895–3,958,580	3.63	1.74	−2.89	284.5–312.5
2018	YR-APR	*QYR-APR-2A*	2A	198	NA	1,106,494–100,058,689	3.52	2.43	5.49	197.5–203.5
	YR-APR	*QYR-APR-7A*	7A	238	86,051,773–91,538,169	7,350,555–3,064,562	5.58	3.85	6.95	237.5–241.5
	YR-APR	*QYR-APR-2B.1*	2B	51	7,726,779	6,050,347–1,275,640	4.06	2.85	5.94	48.5–58.5
	YR-APR	*QYR-APR-3D*	3D	201	612,659,895	3,064,599–100,087,630	10.52	11.43	11.91	193.5–208.5
	YR-APR	*QYR-APR-4D.1*	4D	169	482,004,603 –495,101,244	1,133,723–1,667,202	6.47	6.25	−8.94	163.5–176.5
	YR-APR	*QYR-APR-4D.2*	4D	248	23,317,222–84,937,400	1,201,923–4,909,310	7.48	16.42	14.26	233.5–255.5
	YR-APR	*QYR-APR-5D*	5D	6	88,551,618–340,292,671	7,487,719–2,265,426	3.51	3.99	−7.04	0–16.5
2019	YR-APR	*QYR-APR-1A*	1A	133	17,405,618	991,036–3,934,878	5.71	7.43	9.02	129.5–135.5
	YR-APR	*QYR-APR-1B*	1B	47	670,142,832–679,858,781	1,230,145–4,005,225	8.18	10.20	10.55	46.5–47.5
	YR-APR	*QYR-APR-2B.2*	2B	156	220,348,494–235,259,298	5,577,199–1,054,964	4.90	5.93	8.06	155.5–156.5
	YR-APR	*QYR-APR-4D.1*	4D	165	482,004,603–495,101,244	1,133,723–1,667,202	6.81	8.55	−9.98	163.5–170.5
	YR-APR	*QYR-APR-4D.2*	4D	231	23,317,222–360,914,321	1,001,325–1,201,923	7.08	8.76	9.79	224.5–246.5
Across years	YR-APR	*QYR-APR-1B*	1B	47	670,142,832–679,858,781	1,230,145–4,005,225	3.99	4.10	4.75	46.5–47.5
	YR-APR	*QYR-APR-2B.1*	2B	51	7,726,779	6,050,347–1,275,640	5.94	6.54	6.03	48.5–58.5
	YR-APR	*QYR-APR-2B.2*	2B	153	192,955,204–193,062,823	2,256,116–1,050,655	6.54	6.91	6.17	152.5–154.5
	YR-APR	*QYR-APR-2B.3*	2B	241	781,214,059–797,605,252	1,219,456–1,121,623	3.09	5.40	5.47	229.5–249.5
	YR-APR	*QYR-APR-1D*	1D	50	9,038,548–11,353,637	1,107,347–1,228,408	3.31	3.39	−4.33	35.5–55.5
	YR-APR	*QYR-APR-3D*	3D	211	612,659,895	3,064,599–100,087,630	3.23	12.24	8.23	201.5–218.5
	YR-APR	*QYR-APR-4D.1*	4D	165	482,004,603–495,101,244	1,133,723–1,667,202	5.23	5.58	−5.72	163.5–172.5
	YR-APR	*QYR-APR-4D.2*	4D	240	23,317,222–84,937,400	1,201,923–4,909,310	3.36	6.92	6.18	232.5–255.5
2020	LR-ASR-MLTTH	*QLR-ASR-MLTTH-2A*	2A	307	755,073,581–771,366,169	1,230,056–2,259,439	8.93	6.10	−1.17	299.5–312.5
	LR-ASR-MLTTH	*QLR-ASR-MLTTH-5B*	5B	127	561,598,758	100,058,433–2,267,368	3.07	4.30	−0.99	126.5–127.5
	LR-ASR-MLTTH	*QLR-ASR-MLTTH-6B*	6B	23	378,424,485–692,080,108	1,221,097–7,353,355	5.66	5.81	−1.15	19.5–28.5
	LR-ASR-MLTTH	*QLR-ASR-MLTTH-3D*	3D	105	268,920,926	100,080,288–1,210,613	3.67	0.82	−0.43	97.5–107.5
	LR-ASR-TLTTR	*QLR-ASR-TLTTR-6D*	6D	213	462,479,335–479,773,419	1,040,130–1,063,571	4.99	7.58	−0.37	209.5–215.5
2020	YR-ASR-Pst2	*QYR-ASR-Pst2-3A*	3A	141	25,391,100–25,669,110	4,988,975–2,257,915	3.73	4.75	0.48	139.5–141.5
	YR-ASR-Pst2	*QYR-ASR-Pst2-5A*	5A	93	695,468,777	1,184,257–100,044,187	9.14	14.01	−0.82	89.5–96.5
	YR-ASR-Pst2	*QYR-ASR-Pst2-6D*	6D	126	25,673,385–51,985,701	1,046,205–1,009,547	3.64	5.80	0.54	120.5–135.5

LR_ASR, leaf rust seedling resistance, YR_ASR, yellow rust seedling resistance; LR_APR, leaf rust adult plant resistance; YR_APR, yellow rust adult plant resistance; (leaf rust pathotypes at the seedling stage, THTTQ, TCTTR, TCPTQ, THTTR, MLTTH, and TLTTR; yellow rust pathotypes at the seedling stage, Pst1, Pst2, and Pst3), QTL, quantitative trait locus, cM, centimorgan, LOD, logarithm of odds, PVE, phenotypic variation explained, Add, additive effect; consistent QTLs are highlighted in red font; physical positions are obtained from the reference genome IWGSC RefSeq v2.0.

### Anza population

#### QTLs for yellow and leaf rust APR

For LR APR, a total of six QTLs, i.e., *QLR-APR-4A*, *QLR-APR-5B*, *QLR-APR-1A*, *QLR-APR-2B*, *QLR-APR-3B*, and *QLR-APR-7D*, were identified on different chromosomes at 233 cM, 288 cM, 97 cM, 295−313 cM, 340−342 cM and, 211 cM, respectively. The identified QTLs explained that the percent phenotypic variation ranged from 1.16 (*QLR-APR-3B*) to 15.18 (*QLR-APR-2B*). A maximum of three QTLs was identified on B, two QTLs on A, and one QTL on D subgenomes (Table 4).

For YR APR, 10 QTLs were mapped on 1D, 2A, 4D, 7D, 3A, and 3D at different locations. The 10 identified QTLs, i.e., *QYR-APR-2A.1*, *QYR-APR-2A.2*, *QYR-APR-1D*, *QYR-APR-4D.1*, *QYR-APR-7D*, *QYR-APR-3A.3*, *QYR-APR-4D.2*, *QYR-APR-3A.1*, *QYR-APR-3A.2*, and *QYR-APR-3D*, explained the percent phenotypic variation of 9.94, 10.39, 4.74, 8.88, 7.34, 10.57, 9.36, 10.35, 20.93, and 4.26, respectively. A maximum of five QTLs was observed, each on A and D subgenomes; however, no QTL was identified on the B subgenome.

#### Stable QTLs for APR

A total of six consistent QTLs including three each for LR APR (*QLR-APR-4A*, *QLR-APR-2B*, and *QLR-APR-3B*) and YR APR (*QYR-APR-2A.1*, *QYR-APR-2A.2*, and *QYR-APR-4D.2*) were detected in the Anza population. One QTL, i.e., QLR-APR-2B, was detected during 2019 and 2020, which were flanked between marker intervals 3064426–1125988 at a confidence interval of 287.5 cM–322.5 cM with 15.18% (2019) and 9.41% (2020) PVE, respectively (Table 4). The remaining two consistent QTLs, i.e., *QLR-APR-4A* and *QLR-APR-3B*, were identified in one environment and pooled means. *QLR-APR-4A* was flanked between marker intervals *998,585–1054130* and confidence intervals 230.5 cM–233.5 cM with 8.08 (2018) and 5.76% PVE, respectively (across years), whereas *QLR-APR-3B* was flanked between marker intervals 2257185–4396068 and confidence intervals 325.5 cM–342 cM with 8.08 (2020) and 5.76% PVE, respectively (across years).

Similarly, three consistent/stable QTLs, i.e., *QYR-APR-2A.1*, *QYR-APR-2A.2*, and *QYR-APR-4D.2*, were identified for yellow rust. *QYR-APR-2A.1* was mapped between marker intervals 2260254–3064488 and confidence intervals 96.5 cM–110.5 cM with 9.94% (2018) and 4.07% PVE, respectively (across years); similarly, *QYR-APR-2A.2* was mapped between marker intervals 1212067–1242826 (2018) and 1242826–1230957 (across years) at a confidence interval of 169.5 cM–182.5 cM with PVE of 10.39% (2018) and 4.43% (across years), respectively. The third consistent QTL (*QYR-APR-4D.2*) was mapped between the flanking markers of 1133723–1012563 (2019) and 1012563–3936672 (across years) at a confidence interval of 100.5 cM–110.5 cM with PVE of 9.36% (2019) and 8.56% (across years), respectively.

#### QTLs for yellow and leaf rust ASR

Twelve QTLs, including six each for LR (QLR-ASR-THTTR-7A, QLR-ASR-THTTR-3B, QLR-ASR-TCTTR-6B.1, QLR-ASR-TCTTR-6B.2, QLR-ASR-TCPTQ-2D, and QLR-ASR-TCPTQ-6D) and YR (*QYR-ASR-Pst3-1B*, *QYR-ASR-Pst3-6B.1*, *QYR-ASR-Pst3-6B.2*, *QYR-ASR-Pst3-6B.3*, *QYR-ASR-Pst3-7B*, and *QYR-ASR-Pst1-6D*) were identified in the Anza population. For leaf rust, the highest PVE was reported for *QLR-ASR-TCPTQ-6D* (14.63%) followed by *QLR-ASR-THTTR-7A* (9.07%), *QLR-ASR-THTTR-3B* (6.71%), *QLR-ASR-TCTTR-6B.2* (5.14%), *QLR-ASR-TCTTR-6B.1* (5.07%), and *QLR-ASR-TCPTQ-2D* (3.7%). Similarly, for yellow rust, the highest PVE was reported for *QYR-ASR-Pst1-6D* (8.57%) followed by *QYR-ASR-Pst3-7B* (4.25%), *QYR-ASR-Pst3-6B.1* (4.24%), and *QYR-ASR-Pst3-6B.2* (4.19%).

### Avocet population

#### QTLs for yellow and leaf rust APR

A set of 15 QTLs, including LR (3 QTLs) and YR APR (12 QTLs), were identified in the Avocet population. For leaf rust, three QTLs, i.e., *QLR-APR-5A.1*, *QLR-APR-5A.2*, and *QLR-APR-7A*, flanked between marker intervals *1128503–2262017*, *3064895–3958580*, and *1102911–1309112*, respectively, at a confidence interval of 201.5 cM–202.5 cM, 284.5 cM–312.5 cM, and 86.5 cM–90.5 cM. The highest PVE was reported for *QLR-APR-7A* (11.77%) followed by *QLR-APR-5A.1* (2.79%) and *QLR-APR-5A.2* (2.17%). All the QTLs were mapped only on subgenome A, and there is no representation from subgenomes B and D (Table 5).

For yellow rust APR, 12 QTLs were identified on different chromosomes of all three subgenomes of wheat. The highest PVE was reported for QYR-APR-4D.2 (16.42% in 2018 and 8.76% across years) followed by QYR-APR-3D (12.24% across years and 11.43% in 2018), *QYR-APR-1B* (10.20%), *QYR-APR-4D.1* (8.55%), and QYR-APR-1A (7.43%), and the remaining seven QTLs reported less than 7.0% PVE. The highest QTLs were reported in subgenome D followed by subgenomes B and A.

#### Stable QTLs for APR

A set of seven consistent QTLs including one for LR (*QLR-APR-5A.2*) and six for YR (*QYR-APR-1B*, *QYR-APR-2B.1*, *QYR-APR-2B.2*, *QYR-APR-3D*, *QYR-APR-4D.1*, and *QYR-APR-4D.2*) were identified in the Avocet population. The only consistent QTL, i.e., *QLR-APR-5A.2*, for leaf rust APR was identified in 2018 and across years, which was flanked between marker intervals 3064895–3958580 at a confidence interval of 284.5 cM–312.5 cM. The phenotypic variation explained was comparatively low, with 2.17% during 2018 and 1.74% across the years. A maximum of six consistent QTLs were identified for yellow rust APR. Two consistent QTLs, i.e., *QYR-APR-4D.1* and *QYR-APR-4D.2*, were identified in both the tested environments and also across years, which were flanked between the marker intervals 1133723–1667202 and 1201923–4909310 at confidence intervals 163.5 cM–176.5 cM and 224.5 cM–255.5 cM. Furthermore, these two QTLs reported high PVE which ranges from 5.58% to 8.55% (*QYR-APR-4D.1*) and 6.92% to 16.42% (*QYR-APR-4D.2*) (Table 5). The remaining four consistent QTLs were identified in one environment and also across years. Two QTLs, i.e., *QYR-APR-1B* and *QYR-APR-2B.2*, were identified in 2019 and across years, which were identified in the marker intervals of 1230145–4005225 (*QYR-APR-1B*) and 5577199–1054964 in 2018 and 2256116–1050655 across years (*QYR-APR-2B.2*) at a confidence interval of 46.5 cM–47.5 cM and 152.5 cM–156.5 cM. The remaining two QTLs, i.e., QYR-APR-2B.1 and QYR-APR-3D, were identified in 2018 and across years, which were mapped in the marker intervals of 48.5 cM–58.5 cM and 193.5 cM–218.5 cM, respectively. One QTL, i.e., QYR-APR-3D, also reported high PVE with 11.43% in 2018 and 12.24% across years.

#### QTLs for yellow and leaf rust ASR

A total of eight QTLs including five for LR and three for YR were identified in the Avocet population. For LR ASR, the highest PVE was reported for *QLR-ASR-TLTTR-6D* (7.58%), followed by *QLR-ASR-MLTTH-2A* (6.10%), *QLR-ASR-MLTTH-6B* (5.81%), *QLR-ASR-MLTTH-5B* (4.30%), and *QLR-ASR-MLTTH-3D* (0.82%). For yellow rust, the highest PVE was reported for *QYR-ASR-Pst2-5A* (14.01%), followed by *QYR-ASR-Pst2-6D* (5.80%) and *QYR-ASR-Pst2-3A* (4.75%).

### Putative candidate genes

The putative candidate genes were identified for consistent QTLs with high PVE for leaf and yellow rust APR through *in silico* analysis and are presented in Table 6, and additional details are provided in Supplementary Table S4. The QTL, i.e., *QLR-APR-4A*, located at 707.93–712.15 Mb encodes *cytochrome P450* (TraesCS4A02G443600.1). Similarly, *QLR-APR-3B* located at 22.24–444.82 Mb encodes the *leucine-rich repeat domain superfamily* (TraesCS3B02G043400.1). *QYR-APR-2A.1* located at 683.53–705.99 Mb encodes *zinc-binding ribosomal protein* (TraesCS2A02G458600.1). *QYR-APR-2A.2* located at 17.44–30.33 Mb encodes the *SANT/Myb domain* (TraesCS2A02G069300.1) and *WRKY transcription factor* (TraesCS2A02G043700.1). Other two QTLs, i.e., *QYR-APR-4D.1* encodes the *NAC domain superfamily* (TraesCS4D02G316800.1) and *QYR-APR-2B.2* located at 184.79–235.26 Mb encodes the *nucleotide-sugar transporter* (TraesCS2B02G222600.1).

**TABLE 6:** Putative candidate genes for leaf and yellow rust adult plant resistance.

Trait	QTL name	Population	Physical position (Mb)	TraesID	Putative candidate gene	Molecular function
LR_APR	*QLR-APR-4A*	Almaly x Anza	707.93–712.15	TraesCS4A02G443600.1	*Cytochrome P450*	Monooxygenase activity, iron ion binding, oxidoreductase activity, and heme binding
LR_APR	*QLR-APR-3B*	Almaly x Anza	22.24–444.82	TraesCS3B02G043400.1	*Leucine-rich repeat domain superfamily and protein kinase-like domain superfamily*	Protein kinase activity, nucleotide binding, protein binding, and transferase activity
YR_APR	*QYR-APR-2A.1*	Almaly x Anza	683.53–705.99	TraesCS2A02G458600.1	*Zinc-binding ribosomal protein*	Structural constituent of the ribosome and protein binding
YR_APR	*QYR-APR-2A.2*	Almaly x Anza	17.44–30.33	TraesCS2A02G069300.1	*SANT/Myb domain*	DNA binding
				TraesCS2A02G043700.1	*WRKY transcription factor*	DNA-binding transcription factor activity and sequence-specific DNA binding
YR_APR	*QYR-APR-2B.1*	Almaly x Avocet	NA-5.97	TraesCS2B02G011200.1	*Leucine-rich repeat-containing N-terminal and plant-type*	Protein binding
YR_APR	*QYR-APR-2B.2*	Almaly x Avocet	184.79–235.26	TraesCS2B02G222600.1	*Nucleotide-sugar transporter*	Pyrimidine nucleotide-sugar transmembrane transporter activity
YR_APR	*QYR-APR-4D.1*	Almaly x Avocet	481.88–495.07	TraesCS4D02G316800.1	*NAC domain superfamily*	DNA binding

LR_APR, leaf rust adult plant resistance; YR_APR, yellow rust adult plant resistance.

## Discussion

The present rate of genetic gain is approximately 0.8%–1.2% for the major food crops, including wheat, and in recent years this progress has plateaued. The current rate of annual progress is too short of the 2.4% required to feed approximately 9.5 billion people by 2050 (Krishnappa et al., 2021a; Ray et al., 2012; Ray et al., 2013). Although the genetic progress of crop plants is a continuous process, protection of crop yield from biotic and abiotic stresses is also very important to minimize the crop losses and to have sustainable crop production. Rust (yellow, leaf, and stem) diseases are very important biotic stresses in wheat, which cause substantial crop damage across the globe. Genetic dissection of complex traits through QTL mapping will be helpful in designing the appropriate breeding strategies through MAS (Krishnappa et al., 2021b; Khan et al., 2022). Many of the race-specific/seedling resistance genes identified for all three rusts are from wild relatives, and their direct utilization in breeding programs is hindered due to an undesirable linkage drag associated with resistance locus. Furthermore, the durability of the seedling resistance genes is less compared to APR genes. Hence, to avoid linkage drag and resistance breakdown, plant breeders showed much interest in molecular studies in elite genetic backgrounds (Tehseen et al., 2022).

Phenotyping of 206 RILs from the Avocet and 160 RILs from Anza population suggests the presence of a wide variability of resistance to both the rusts, APR and ASR. Previously, a similar kind of broad variability was observed for wheat leaf rust (Rollar et al., 2021). A high broad sense heritability of approximately 0.85 and above was recorded for all the traits including leaf and yellow rust APR and leaf and yellow rust ASR in both populations. A similar range of broad sense heritability was also observed in previous reports (Rollar et al., 2021; Gao et al., 2016). The magnitude of correlations between leaf and yellow rust APR was relatively low, although a slight positive correlation was observed in the Anza population.

Overall, 51 QTLs in Anza (28 QTLs) and Avocet populations (23 QTLs) were identified. The 28 QTLs mapped in the Anza population represent leaf rust APR (6 QTLs), yellow rust APR (10 QTLs), leaf rust ASR (6 QTLs), and yellow rust ASR (6 QTLs). The 23 QTLs identified in the Avocet population represent leaf rust APR (3 QTLs), yellow rust APR (12 QTLs), leaf rust ASR (5 QTLs), and yellow rust ASR (3 QTLs). A maximum of 22 QTLs were mapped for yellow rust APR, followed by leaf rust ASR (11 QTLs), leaf rust APR, and ASR (6 QTLs each). Similarly, disease-wise representation of QTLs includes 31 QTLs for yellow rust and 20 QTLs for leaf rust. QTLs were equally distributed among the three subgenomes with 17 QTLs each. The maximum number of QTLs were mapped on chromosome 6B (6 QTLs) followed by 2A, 2B, 3A, 4D, and 6D (4 QTLs each); 3D, 5A, and 7A (3 QTLs each); 1A, 1B, 1D, 3B, 5B, and 7D (2 QTLS each); and 2D, 4A, 5D, and 7B (1 QTL each).

Nine QTLs were detected for leaf rust APR on eight chromosomes. Two major QTLs (*QLR-APR-2B* and *QLR-APR-7A*) explained more than 11.0% PVE. Previously, one leaf rust APR gene, i.e., *Lr 34/Yr18*, was identified on the 7D chromosome on the Lalbahadur bread wheat cultivar through single-chromosome substitutions from the Parula cultivar, a source of *Lr34/Yr18* (Lagudah et al., 2006). Similarly, the 7D chromosome is important as many of the QTLs were detected previously on this chromosome (Gao et al., 2016; Li et al., 2016; Zhang et al., 2017; Bokore et al., 2020; Gebrewahid et al., 2020). We also detected two QTLs in 7D, namely, QLR-APR-7D and QYR-APR-7D, that had PVE of 4.81% and 7.34%, respectively. Similarly, several previous studies also reported QTLs for leaf rust APR on the same chromosomes 1A, 2B, 3B, 4A, 5A, 5B, and 7A at different locations (Gao et al., 2016; Li et al., 2016; Lan et al., 2017; Zhang et al., 2017; Zhang et al., 2019; Bokore et al., 2020; Gebrewahid et al., 2020; Rauf et al., 2022). For leaf rust seedling resistance, 11 QTLs were identified in both Anza and Avocet populations. Three major QTLs, i.e., *QLR-ASR-TCPTQ-6D*, *QLR-ASR-TLTTR-6D*, and *QLR-ASR-THTTR-7A*, had PVE of 14.63%, 7.58%, and 9.07%, respectively, and mapped at marker intervals 100,023455–1091595, 1040130–1063571, and 1111941–1125395 and confidence intervals 216.5 cM–228.5 cM, 209.5 cM–215.5 cM, and 137.5 cM–144.5 cM, respectively. Previously, several leaf rust ASR genes including *Lr2a*, *Lr3*, *Lr3a*, *Lr9*, *Lr11*, *Lr15*, *Lr17*, *Lr18*, *Lr20*, *Lr22a*, *Lr32*, *Lr36*, *Lr37*, *Lr38*, *Lr39*, *Lr41*, *Lr45*, *Lr47*, *Lr52*, *Lr53*, *Lr59*, *65*, *Lr77*, *Lr79*, and *Lr80* were identified on chromosomes 2A, 2D, 3B, 3D, 5B, 6B, 6D, and 7A, respectively (Kumar et al., 2022); whereas in our study, 11 QTLs were mapped on the same chromosomes. Furthermore, previously, few leaf rust ASR QTLs were identified on the same chromosomes at different locations (Gao et al., 2016; Li et al., 2016; Zhang et al., 2019; Delfan et al., 2023). A maximum of 22 QTLs were found on different chromosomes covering all three subgenomes for yellow rust APR. A set of eight major yellow rust ASR QTLs had PVE ≥10.0%, with the highest variation explained by *QYR-APR-3A.2* (20.93%) followed by *QYR-APR-4D.2* (16.42%), *QYR-APR-3D* (12.24%), *QYR-APR-3D* (11.43%), *QYR-APR-3A.3* (10.57%), *QYR-APR-2A.2* (10.39%), *QYR-APR-3A.1* (10.35%), and *QYR-APR-1B* (10.20%). Three yellow rust APR genes, i.e., *Yr49* and *Yr71* on 3D and *Yr75* on 7A chromosomes, were reported in previous studies (Jamil et al., 2020). Previously, a few yellow rust APR QTLs were also identified on the same chromosomes in different mapping populations and marker systems (Lan et al., 2017; Yuan et al., 2018; Long et al., 2019; Zhang et al., 2019; Gebrewahid et al., 2020; Farzand et al., 2021; Cheng et al., 2022; Tehseen et al., 2022). In our study, nine yellow rust ASR QTLs on chromosomes 1B, 3A, 5A, 6B, 6D, and 7B were identified. Out of nine QTLs, two major QTLs explained ≥8.0% PVE, with the highest variation explained by *QYR-ASR-Pst2-5A* (14.01%) followed by *QYR-ASR-Pst1-6D* (8.57%).

Detection and validation of consistent QTLs in multiple environments are critical for their effective utilization through marker-based breeding approaches (Krishnappa et al., 2023). In this direction, Anza population was phenotyped for 3 consecutive years (2018, 2019, and 2020), whereas Avocet population was tested during 2 consecutive years (2018 and 2019). As a result, 13 consistent QTLs including nine QTLs for yellow rust APR (*QYR-APR-2A.1*, *QYR-APR-2A.2*, *QYR-APR-4D.2*, *QYR-APR-1B*, *QYR-APR-2B.1*, *QYR-APR-2B.2*, *QYR-APR-3D*, *QYR-APR-4D.1*, and *QYR-APR-4D.2*) and four QTLs for leaf rust APR (*QLR-APR-4A*, *QLR-APR-2B*, *QLR-APR-3B*, and *QLR-APR-5A.2*) were identified. Stable QTLs are promising candidates for further validation in diverse backgrounds and exploitation in marker-assisted selection. Previously, Gebrewahid et al. (2020) reported QTLs on chromosomes 2BS and 5AL conferred resistance to both YR and LR and proposed that *QYr.hebau-5AL/QLr.hebau-5AL* are likely to be novel. Zhang et al. (2019) identified QTLs (*QLr.hebau-5AL/QYr.hebau-5AL*) on chromosome 5AL conferred resistance to both rusts; they are likely to be new QTLs. Bokore et al. (2023) reported that the wheat cultivar Carberry contributed QTLs conferring LR APR on 2B (two loci, i.e., *QLr.spa-2B.2* and *QLr.spa-2B.1*) and 5A (*QLr.spa-5A*). Kumar et al. (2020) identified three distinct loci revealed on chromosomes 2B (*QLr.ramp-2B.7* and *QLr.ramp-2B.8*) and 5A (*QLr.ramp-5A*) to be associated with LR-APR. QTLs associated with stripe rust resistance APR were identified on chromosome 3D (Ye et al., 2019; Habib et al., 2020). A significant association of IWA5707 and other linked SNPs (IWA6277, IWA5375, and IWA5766) was detected on the short arm of chromosome 4D at 25.7 cM. Muleta et al. (2017) and Forrest et al. (2014) reported a significant association of IWA5707, IWA5375, and IWA5766 on chromosome 4D with resistance to YR. A putatively new QTL, linked to LR APR, was identified on chromosome 4D (Rollar et al., 2021). Mapping results identified QTL-conferring APR to stripe rust resistance also on 4DL (Zhang et al., 2022). One QTL, i.e., *QLr.cimmyt-5A* associated with APR LR, was mapped on the long arm of chromosome 5A and closely linked to *Vrn-A1* at 587.0 Mb (Rosewarne et al., 2012).

The putative candidate genes for the QTLs are provided in Table 6; Supplementary Table S4. For instance, *QLR-APR-4A* encodes *cytochrome P450* (TraesCS4A02G443600.1) found to have a role in rust resistance, and transcription profiling suggests that transcripts encoding *cytochrome P450* were upregulated (Hulbert et al., 2007; Manickavelu et al., 2010; Wu et al., 2019) during rust pathogen infection. Similarly, *QLR-APR-3B* encodes the *leucine-rich repeat domain superfamily* (TraesCS3B02G043400.1) that is crucial for wheat rust resistance. The resistant hexaploid wheat variety Thatcher *Lr10* encodes a *nucleotide-binding site* (NBS) and *leucine-rich repeat* (LRR), which play a role in wheat leaf rust resistance (Feuillet et al., 2003). Most R-genes encode intracellular *nucleotide-binding leucine-rich-repeat receptors (NBS-LRRs)*, which play a key role in wheat rust resistance (Hao et al., 2016; Basnet et al., 2022). Inactivation of the wheat Ser/Thr kinase gene, i.e., *Puccinia striiformis*-induced protein kinase 1 (TaPsIPK1), results in broad-spectrum resistance to *Pst* races (Wang et al., 2022). The QTL, i.e., *QYR-APR-2A.1,* encodes *zinc-binding ribosomal protein* (TraesCS2A02G458600.1). Wheat zinc finger protein *TaLSD1* regulates a hypersensitive response in plants, thereby conferring stripe rust resistance (Guo et al., 2013). *QYR-APR-2A.2* encodes the *SANT/Myb domain* (TraesCS2A02G069300.1). TFs including *Zn finger-binding proteins*, *SANT/Myb domains*, *NAC domain*, and *BTF3* play an important role in imparting stripe rust resistance (Jan et al., 2021). *QYR-APR-2A.2* encodes the *WRKY transcription factor* (TraesCS2A02G043700.1). Wang et al. (2020) and Wang et al. (2016) reported the role of *TaWRKY70* in YR resistance, particularly during the seedling stage. Furthermore, transgenic overexpression of barley WRKY genes, namely, *HvWRKY6* and *HvWRKY70*, confers YR resistance (Li et al., 2020). *QYR-APR-2B.2* encodes the *nucleotide-sugar transporter* (TraesCS2B02G222600.1), sugar transporters like *TaSTP6* (Huai et al., 2019), *TaSTP13* (Huai et al., 2020), and *PsHXT1* (Chang et al., 2020) are essential for the pathogenicity of the wheat rust pathogen, and it promotes wheat susceptibility to stripe rust. Another QTL *QYR-APR-4D.1* encodes the *NAC domain superfamily* (TraesCS4D02G316800.1), and wheat *NAC Transcription factors* like *TaNAC069* (Xu et al., 2022) and *TuNAC69* (Zhang et al., 2021) regulate leaf and stripe rust resistance, respectively. In crop plants, the majority of the disease-resistance genes is race-specific and contains the NBS and LRR domains. These resistant genes or QTLs are believed to be regulated by NBS domains through signal transduction, and the specific sites of corresponding pathogen virulence genes are recognized by LRR domains (Gill et al., 2015). Some of the stable QTLs like *QLR-APR-2B* (Anza population) and *QYR-APR-2B.2* (Avocet population) were found to encode *nucleotide-binding domains* which play a role in disease resistance. Similarly, other stable QTLs such as *QYR-APR-4D.2* (Anza population) and *QYR-APR-4D.1* (Avocet population) encode important putative genes like the *zinc finger C2H2 superfamily*, which play an important role in disease resistance in plants (Guo et al., 2013). Therefore, stable QTLs which encode the same putative candidate genes could be potential candidate genomic regions for further functional validation.

## Conclusion

The study with two RIL populations derived from a cross between Almaly × Anza (160 RILs) and Almaly × Avocet S (206 RILs) suggested the presence of wide variability for yellow and leaf rust APR and ASR. We identified a set of 13 consistent QTLs including yellow rust APR (9 QTLs) and leaf rust APR (4 QTLs). Among them, *QLR-APR-2B* and *QYR-APR-4D.2* from the Anza population and *QLR-APR-5A.2*, *QYR-APR-4D.1*, *QYR-APR-4D.2*, and *QYR-APR-3D* from the Avocet population are important candidates to target for further validation and deployment in LR and YR resistance breeding. Several putative candidate genes were identified in this study; mainly, zinc finger proteins, DNA-binding pseudobarrel domain superfamily, and NAC domain superfamily with the associated functions in the resistance mechanism of leaf and yellow rust were identified. The functional characterization of these candidate genes will provide greater applicability of this study in rust resistance breeding.

## Data Availability

The phenotypic and genotypic datasets used in this study are available as Supplementary Material (Supplementary Table S1, Supplementary Table S2). Further, they were submitted to the open access repository “DRYAD” and they will be accessible through the link https://doi.org/10.5061/dryad.3bk3j9krn.

## References

[B1] Aktar-Uz-Zaman M. Tuhina-Khatun M. Hanafi M. M. Sahebi M. (2017). Genetic analysis of rust resistance genes in global wheat cultivars: an overview. Biotechnol. Biotechnol. Equip. 31 (3), 431–445. 10.1080/13102818.2017.1304180

[B2] Ali S. Gladieux P. Leconte M. Gautier A. Justesen A. F. Hovmoller M. S. (2014). Origin, migration routes and worldwide population genetic structure of the wheat yellow rust pathogen *Puccinia Striiformis f.* sp*. Tritici* . PLoS Pathog. 10, e1003903. 10.1371/journal.ppat.1003903 24465211 PMC3900651

[B3] Basnet B. Juliana P. Bhattarai K. Upreti U. (2022). A review on major rust resistance gene and amino acid changes on wheat (*Triticum aestivum L*). Adv. Agric. 4, 1–11. 10.1155/2022/7419326

[B4] Bokore F. E. Knox R. E. Cuthbert R. D. Pozniak C. J. McCallum B. D. N’Diaye A. (2020). Mapping quantitative trait loci associated with leaf rust resistance in five spring wheat populations using single nucleotide polymorphism markers. PLoS One 15 (4), e0230855. 10.1371/journal.pone.0230855 32267842 PMC7141615

[B5] Chang Q. Lin X. Yao M. Liu P. Guo J. Huang L. (2020). Hexose transporter PsHXT1-mediated sugar uptake is required for pathogenicity of wheat stripe rust. Plant Biotechnol. J. 18 (12), 2367–2369. 10.1111/pbi.13398 32386262 PMC7680534

[B6] Chen X. (2013). High-temperature adult-plant resistance, key for sustainable control of stripe rust. Am. J. Plant Sci. 4, 608–627. 10.4236/ajps.2013.43080

[B7] Cheng B. Gao X. Cao N. Ding Y. Chen T. Zhou Q. (2022). QTL mapping for adult plant resistance to wheat stripe rust in M96-5 × Guixie 3 wheat population. J. Appl. Genet. 63, 265–279. 10.1007/s13353-022-00686-z 35338429 PMC8979893

[B8] Ciechanowskaa I. Semagn K. McCallum B. Randhawa H. Strenzke K. Dhariwal R. (2022). Quantitative trait locus mapping of rust resistance and agronomic traits in spring wheat. Can. J. Plant Sci. 102, 1139–1150. 10.1139/CJPS-2022-0023

[B9] Delfan S. Bihamta M. R. Dadrezaei S. T. Abbasi A. Alipour H. (2023). Exploring genomic regions involved in bread wheat resistance to leaf rust at seedling/adult stages by using GWAS analysis. BMC Genom 24, 83. 10.1186/s12864-022-09096-1 PMC9945389 36810004

[B10] Dreisigacker S. Shewayrga H. Crossa J. Arief V. N. DeLacy I. H. Singh R. P. (2012). Genetic structures of the CIMMYT international yield trial targeted to irrigated environments. Mol. Breed. 29, 529–541. 10.1007/s11032-011-9569-7

[B11] Edet O. U. Gorafi Y. S. A. Nasuda S. Tsujimoto H. (2018). DArTseq-based analysis of genomic relationships among species of tribe Triticeae. Sci. Rep. 8, 16397. 10.1038/s41598-018-34811-y 30401925 PMC6219600

[B12] Ellis J. G. Lagudah E. S. Spielmeyer W. Dodds P. N. (2014). The past, present and future of breeding rust resistant wheat. Front. Plant Sci. 5, 641. 10.3389/fpls.2014.00641 25505474 PMC4241819

[B13] Farzand M. Dhariwal R. Hiebert C. W. Spaner D. Randhawa H. S. (2021). Mapping quantitative trait loci associated with stripe rust resistance from the Canadian wheat cultivar ‘AAC Innova. Can. J. Plant Pathol. 43, S227–S241. 10.1080/07060661.2021.1982011

[B14] Feuillet C. Travella S. Stein N. Albar L. Nublat A. Keller B. (2003). Map-based isolation of the leaf rust disease resistance gene *Lr10* from the hexaploid wheat (*Triticum aestivum L.)* genome. PNAS U. S. A. 100 (25), 15253–15258. 10.1073/pnas.2435133100 PMC299976 14645721

[B15] Flor H. H. (1971). Current status of the gene-for-gene concept. Annu. Rev. Phytopathol. 9, 275–296. 10.1146/annurev.py.09.090171.001423

[B16] Forrest K. Pujol V. Bulli P. Pumphrey M. Wellings C. Herrera-Foessel S. (2014). Development of a SNP marker assay for the *Lr67* gene of wheat using a genotyping by sequencing approach. Mol. Breed. 34, 2109–2118. 10.1007/s11032-014-0166-4

[B17] Gao L. Turner M. K. Chao S. Kolmer J. Anderson J. A. (2016). Genome wide association of seedling and adult plant resistance in elite spring wheat breeding lines. PLoS One 11, e0148671. 10.1371/journal.pone.0148671 26849364 PMC4744023

[B18] Gassner G. Straib W. (1932). Untersuchungen Über die Infektions bedingungen von *Puccinia glumarum* und *Puccinia graminis* . Arb. Biol. Reichsanst.Land-Forst- wirtsch Berlin-Dahlem. 16 (4), 609–629.

[B19] Gebrewahid T. W. Zhang P. Zhou Y. Yan X. Xia X. He Z. (2020). QTL mapping of adult plant resistance to stripe rust and leaf rust in a Fuyu 3/Zhengzhou 5389 wheat population. Crop J. 8 (4), 655–665. 10.1016/j.cj.2019.09.013

[B20] Gill U. S. Lee S. Mysore K. S. (2015). Host versus non-host resistance: distinct wars with similar arsenals. Phytopathology 105 (5), 580–587. 10.1094/phyto-11-14-0298-rvw 25626072

[B21] Godoy J. G. Rynearson S. Chen X. Pumphrey M. (2018). Genome-wide association mapping of loci for resistance to stripe rust in north American elite spring wheat germplasm. Phytopathology 108, 234–245. 10.1094/PHYTO-06-17-0195-R 28952421

[B22] Guo J. Bai P. Yang Q. Liu F. Wang X. Huang L. (2013). Wheat zinc finger protein TaLSD1, a negative regulator of programmed cell death, is involved in wheat resistance against stripe rust fungus. Plant. Physiol. biochem. 71, 164–172. 10.1016/j.plaphy.2013.07.009 23933226

[B23] Habib M. Awan F. S. Sadia B. Zia M. A. (2020). Genome-wide association mapping for stripe rust resistance in Pakistani spring wheat genotypes. Plants 9, 1056. 10.3390/plants9091056 32824927 PMC7570266

[B24] Hao Y. Wang T. Wang K. Wang X. Fu Y. Huang L. (2016). Transcriptome analysis provides insights into the mechanisms underlying wheat plant resistance to stripe rust at the adult plant stage. PLoS One 11 (3), e0150717. 10.1371/journal.pone.0150717 26991894 PMC4798760

[B25] Herrera-Foessel S. A. Singh R. P. Huerta-Espino J. Crossa J. Yuen J. Djurle A. (2006). Effect of leaf rust on grain yield and yield traits of durum wheats with race-specifc and slow-rusting resistance to leaf rust. Plant Dis. 10.1094/PD-90-1065 30781301

[B26] Huai B. Yang Q. Qian Y. Qian W. Kang Z. Liu J. (2019). ABA-Induced sugar transporter TaSTP6 promotes wheat susceptibility to stripe rust. Plant Physiol. 181 (3), 1328–1343. 10.1104/pp.19.00632 31540949 PMC6836835

[B27] Huai B. Yang Q. Wei X. Pan Q. Kang Z. Liu J. (2020). TaSTP13 contributes to wheat susceptibility to stripe rust possibly by increasing cytoplasmic hexose concentration. BMC Plant Biol. 20 (1), 49. 10.1186/s12870-020-2248-2 32000681 PMC6993525

[B28] Huerta-Espino J. Singh R. P. Germán S. Mccallum B. D. Park R. F. Chen W. Q. (2011). Global status of wheat leaf rust caused by *Puccinia triticina* . Euphytica 179, 143–160. 10.1007/s10681-011-0361-x

[B29] Hulbert S. H. Bai J. Fellers J. P. Pacheco M. G. Bowden R. L. (2007). Gene expression patterns in near isogenic lines for wheat rust resistance gene *Lr34/Yr18* . Phytopathology 97 (9), 1083–1093. 10.1094/PHYTO-97-9-1083 18944173

[B30] International Wheat Genome Sequencing Consortium (IWGSC) (2018). Shifting the limits in wheat research and breeding using a fully annotated reference genome. Science 361 (6403). 10.1126/science.aar7191 30115783

[B31] Jamil S. Shahzad R. Ahmad S. Fatima R. Zahid R. Anwar M. (2020). Role of genetics, genomics, and breeding approaches to combat stripe rust of wheat. Front. Nutr. 7, 580715. 10.3389/fnut.2020.580715 33123549 PMC7573350

[B32] Jan I. Saripalli G. Kumar K. Kumar A. Singh R. Batra R. (2021). Meta-QTLs and candidate genes for stripe rust resistance in wheat. Sci. Rep. 11 (1), 22923. 10.1038/s41598-021-02049-w 34824302 PMC8617266

[B33] Johnson R. Stubbs R. W. Fuchs E. Chamberlain N. H. (1972). Nomenclature for physiological races of *Puccinia striiformis* infecting wheat. Trans. Br. Mycol. Soc. 58, 475–480. 10.1016/S0007-1536(72)80096-2

[B34] Jones J. D. G. Dangl J. L. (2006). The plant immune system. Nature 444, 323–329. 10.1038/nature05286 17108957

[B35] Khan H. Krishnappa G. Kumar S. Mishra C. N. Krishna H. Devate N. B. (2022). Genome-wide association study for grain yield and component traits in bread wheat (*Triticum aestivum L*.). Front. Genet. 13, 982589. 10.3389/fgene.2022.982589 36092913 PMC9458894

[B36] Koishybaev M. K. Zhanarbekova A. B. Kokhmetova A. M. Rsaliev Sh. S. (2010). Genetic study of wheat resistance to leaf rust. Izvestiya NAS RK. Ser. Biol. Med. 6, 10–15.

[B37] Kokhmetova A. Madenova M. Purnhauser L. Kampitova G. Urazaliev R. Yessimbekova M. (2016). Identification of leaf rust resistance genes in wheat cultivars produced in Kazakhstan. Cereal Res. Commun. 44, 240–250. 10.1556/0806.43.2015.056

[B38] Kokhmetova A. Rsaliyev A. Malysheva A. Atishova M. Kumarbayeva M. Keishilov Z. (2021a). Identification of stripe rust resistance genes in common wheat cultivars and breeding lines from Kazakhstan. Plants 10, 2303. 10.3390/plants10112303 34834666 PMC8619625

[B39] Kokhmetova A. Rsaliyev S. Atishova M. Kumarbayeva M. Malysheva A. Keishilov Z. (2021b). Evaluation of wheat germplasm for resistance to leaf rust (Puccinia triticina) and identification of the sources of Lr resistance genes using molecular markers. Plants 10 (7), 1484. 10.3390/plants10071484 34371688 PMC8309318

[B40] Kokhmetova A. Sharma R. Rsaliyev S. Galymbek K. Baymagambetova K. Ziyaev Z. (2018). Evaluation of Central Asian wheat germplasm for stripe rust resistance. Plant Genet. Resour. 16, 178–184. 10.1017/S1479262117000132

[B41] Kolmer J. A. Bajgain P. Rouse M. N. Li J. Zhang P. (2023). Mapping and characterization of the recessive leaf rust resistance gene *Lr83* on wheat chromosome arm 1DS. Theor. Appl. Genet. 136 (5), 115. 10.1007/s00122-023-04361-7 37083869

[B42] Kolmer J. A. Kabdulova M. G. Mustafina M. A. Zhemchuzhina N. S. Dubovoy V. (2014). Russian populations of *Puccinia triticina* in distant regions are not differentiated for virulence and molecular genotype. Plant Pathol. 64, 328–336. 10.1111/ppa.12248

[B43] Kolmer J. A. Ordonez M. E. (2007). Genetic differentiation of *Puccinia triticina* populations in central Asia and the caucasus. Phytopathology 97, 1141–1149. 10.1094/PHYTO-97-9-1141 18944179

[B44] Kolmer J. A. A. (2015). QTL on chromosome 5BL in wheat enhances leaf rust resistance of Lr46. Mol. Breed. 35, 74. 10.1007/s11032-015-0274-9

[B45] Kosambi D. D. (1943). The estimation of map distance from recombination values. Ann. Eugen. 12, 172–175. 10.1111/j.1469-1809.1943.tb02321.x

[B46] Krattinger S. G. Sucher J. Selter L. L. Chauhan H. Zhou B. Tang M. Z. (2016). The wheat durable, multipathogen resistance gene *Lr34* confers partial blast resistance in rice. Plant Biotechnol. 14, 1261–1268. 10.1111/pbi.12491 PMC11388880 26471973

[B47] Krishnappa G. Khan H. Krishna H. Devate N. B. Kumar S. Mishra C. N. (2023). Genome-wide association study for grain protein, thousand kernel weight, and normalized difference vegetation index in bread wheat (*Triticum aestivum* L.). Genes 14, 637. 10.3390/genes14030637 36980909 PMC10048783

[B48] Krishnappa G. Rathan N. D. Sehgal D. Ahlawat A. K. Singh S. K. Singh S. K. (2021b). Identification of novel genomic regions for biofortification traits using an SNP marker-enriched linkage map in wheat (*Triticum aestivum* L.). Front. Nutr. 8, 669444. 10.3389/fnut.2021.669444 34211996 PMC8239140

[B49] Krishnappa G. Savadi S. Tyagi B. S. Singh S. K. Mamrutha H. M. Kumar S. (2021a). Integrated genomic selection for rapid improvement of crops. Genomics 113, 1070–1086. 10.1016/j.ygeno.2021.02.007 33610797

[B50] Kthiri D. Loladze A. N’Diaye A. Nilsen K. T. Walkowiak S. Dreisigacker S. (2019). Mapping of genetic loci conferring resistance to leaf rust from three globally resistant durum wheat sources. Front. Plant Sci. 10, 1247. 10.3389/fpls.2019.01247 31649708 PMC6792298

[B51] Kumar D. Kumar A. Chhokar V. Gangwar O. P. Bhardwaj S. C. Sivasamy M. (2020). Genome-Wide Association Studies in diverse spring wheat panel for stripe, stem, and leaf rust resistance. Front. Plant Sci. 11, 748. 10.3389/fpls.2020.00748 32582265 PMC7286347

[B52] Kumar K. Jan I. Saripalli G. Sharma P. K. Mir R. R. Balyan H. S. (2022). An update on resistance genes and their use in the development of leaf rust resistant cultivars in wheat. Front. Genet. 13, 816057. 10.3389/fgene.2022.816057 35432483 PMC9008719

[B53] Lagudah E. S. McFadden H. Singh R. P. Huerta-Espino J. Bariana H. S. Spielmeyer W. (2006). Molecular genetic characterization of the *Lr34/yr18* slow rusting resistance gene region in wheat. Theor. Appl. Genet. 114 (1), 21–30. 10.1007/s00122-006-0406-z 17008991

[B54] Lan C. Hale I. L. Herrera-Foessel S. A. Basnet B. R. Randhawa M. S. Huerta-Espino J. (2017). Characterization and mapping of leaf rust and stripe rust resistance loci in hexaploid wheat lines UC1110 and PI610750 under Mexican environments. Front. Plant Sci. 8, 1450. 10.3389/fpls.2017.01450 28878791 PMC5573434

[B55] Li C. Bai G. Carver B. F. Chao S. Wang Z. H. (2016). Mapping quantitative trait loci for plant adaptation and morphology traits in wheat using single nucleotide polymorphisms. Euphytica 208, 299–312. 10.1007/s10681-015-1594-x

[B56] Li H. Li Z. Wang J. (2008). Inclusive composite interval mapping (ICIM) for digenic epistasis of quantitative traits in biparental populations. Theor. Appl. Genet. 116, 243–260. 10.1007/s00122-007-0663-5 17985112

[B57] Li H. Wu J. Shang X. Geng M. Gao J. Zhao S. (2020). WRKY transcription factors shared by BTH-induced resistance and NPR1-mediated acquired resistance improve broad-spectrum disease resistance in wheat. Mol. Plant. Microbe. Interact. 33 (3), 433–443. 10.1094/MPMI-09-19-0257-R 31821091

[B58] Liu L. Wang M. N. Feng J. Y. See D. R. Chao S. M. Chen X. M. (2018). Combination of all-stage and high-temperature adult-plant resistance QTL confers high-level, durable resistance to stripe rust in winter wheat cultivar Madsen. Theor. Appl. Genet. 131 (9), 1835–1849. 10.1007/s00122-018-3116-4 29797034

[B59] Long D. L. Kolmer J. A. A. (1989). North American system of nomenclature for *Puccinia recondita f. sp. tritici* . Phytopathology 79, 525–529.

[B60] Long L. Yao F. Yu C. Ye X. Cheng Y. Wang Y. (2019). Genome-wide association study for adult-plant resistance to stripe rust in Chinese wheat landraces (*Triticum aestivum* L.) from the yellow and huai river valleys. Front. Plant Sci. 10, 596. 10.3389/fpls.2019.00596 31156668 PMC6532019

[B61] Mains E. B. Jackson H. S. (1926). Physiologic specialization in the leaf rust of wheat, *Puccinia triticina Erikss* . Phytopathology 16, 89–120.

[B62] Manickavelu A. Kawaura K. Oishi K. Shin-I T. Kohara Y. Yahiaoui N. (2010). Comparative gene expression analysis of susceptible and resistant near-isogenic lines in common wheat infected by *Puccinia triticina* . DNA Res. 4, 211–222. 10.1093/dnares/dsq009 PMC2920755 20360266

[B63] McIntosh R. Dubcovsky J. Rogers W. Xia X. Raupp W. (2020). Catalogue of gene symbols for wheat: 2020 supplement. Ann. Wheat Newsl. 66, 109–128.

[B64] McIntosh R. A. Dubcovsky J. Rogers W. J. Morris C. Appels R. Xia X. C. (2016). Catalogue of gene symbols for wheat: 2015–2016 supplement. Available at: https://shigen.nig.ac.jp/wheat/komugi/genes/macgene/supplement2015.pdf.

[B65] McIntosh R. A. Wellings C. R. Park R. F. (1995). Wheat rusts: an atlas of resistance genes. Australia: CSIRO.

[B66] Morgounov A. Tufan H. A. Sharma R. Akin B. Bagci A. Braun H. J. (2013). Global incidence of wheat rusts and powdery mildew during 1969-2010 and durability of resistance of winter wheat variety Bezostaya 1. Eur. J. Plant Pathol. 132, 323–340. 10.1007/s10658-011-9879-y

[B67] Muleta K. T. Bulli P. Rynearson S. Chen X. Pumphrey M. (2017). Loci associated with resistance to stripe rust (*Puccinia striiformis* F. Sp*. Tritici*) in a core collection of spring wheat (*Triticum aestivum*). PLoS One 12, e0179087. 10.1371/journal.pone.0179087 28591221 PMC5462451

[B68] Peterson R. F. Campbell A. B. Hannah A. E. A. (1948). Diagrammatic scale for estimating rust intensity on leaves and stems of cereals. Canad. J. Res. 26, 496–500. 10.1139/cjr48c-033

[B69] Randhawa H. Puchalski B. J. Frick M. Goyal A. Despins T. Graf R. J. (2012). Stripe rust resistance among western Canadian spring wheat and triticale varieties. Can. J. Plant. Sci. 92 (4), 713–722. 10.4141/cjps2011-252

[B70] Rathan N. D. Krishnappa G. Singh A. M. Govindan V. (2023). Mapping QTL for phenological and grain-related traits in a mapping population derived from high-zinc-biofortified wheat. Plants 12 (1), 220. 10.3390/plants12010220 36616350 PMC9823887

[B71] Rauf Y. Lan C. Randhawa M. Singh R. P. Huerta‐Espino J. Anderson J. A. (2022). Quantitative trait loci mapping reveals the complexity of adult plant resistance to leaf rust in spring wheat ‘Copio. Crop Sci. 62 (3), 1037–1050. 10.1002/csc2.20728

[B72] Ray D. K. Mueller N. D. West P. C. Foley J. A. (2013). Yield trends are insufficient to double global crop production by 2050. PLoS ONE 8 (6), e66428. 10.1371/journal.pone.0066428 23840465 PMC3686737

[B73] Ray D. K. Ramankutty N. Mueller N. D. WestFoley P. C. J. A. (2012). Recent patterns of crop yield growth and stagnation. Nat. Commun. 3, 1293. 10.1038/ncomms2296 23250423

[B74] Roelfs A. P. Singh R. P. Saari E. E. (1992). Rust diseases of wheat: Concepts and methods of disease management. Mexico, D.F.: CIMMYT, 81.

[B75] Rollar S. Serfling A. Geyer M. Hartl L. Mohler V. Ordon F. (2021). QTL mapping of adult plant and seedling resistance to leaf rust (*Puccinia triticina Eriks*.) in a multiparent advanced generation intercross (MAGIC) wheat population. Theor. Appl. Genet. 134 (1), 37–51. 10.1007/s00122-020-03657-2 33201290 PMC7813716

[B76] Rosewarne G. M. Singh R. P. Huerta-Espino J. Herrera-Foessel S. A. Forrest K. L. Hayden M. J. (2012). Analysis of leaf and stripe rust severities reveals pathotype changes and multiple minor QTLs associated with resistance in an Avocet × Pastor wheat population. Theor. Appl. Genet. 124 (7), 1283–1294. 10.1007/s00122-012-1786-x 22274764

[B77] Sansaloni C. Petroli C. Jaccoud D. Carling J. Detering F. (2011). Diversity arrays technology (DArT) and next-generation sequencing combined: genomewide, high throughput, highly informative genotyping for molecular breeding of Eucalyptus. BMC Proc. 5 (54). 10.1186/1753-6561-5-S7-P54

[B78] Schachtel G. A. Dinoor A. Herrmann A. Kosman E. (2012). Comprehensive evaluation of virulence and resistance data: a new analysis Tool. Plant Dis. 96, 1060–1063. 10.1094/PDIS-02-12-0114-SR 30727207

[B79] Sharma R. C. Morgounov A. Akin B. Bespalova L. Lang L. Litvinenko M. (2014). Winter wheat East European regional yield trial: identification of superior genotypes and characterization of environments. Crop Sci. 54, 2469–2480. 10.2135/cropsci2014.01.0028

[B80] Sharma-Poudyal D. Chen X. M. Wan A. M. Zhan G. M. Kang Z. S. Cao S. Q. (2013). Virulence characterization of international collections of the wheat stripe rust pathogen, *Puccinia striiformis* f. sp. *tritici* . Plant Dis. 97, 379–386. 10.1007/s00122-023-04374-2 10.1007/s00122-023-04374-2 30722363

[B81] Singh R. P. Huerta-Espino J. Rajaram S. (2000). Achieving near immunity to leaf and stripe rusts in wheat by combining slow rusting resistance genes. Acta Phytopathologica Entomologica Hung. 35, 133–139.

[B82] Singh S. Bowden R. L. (2011). Molecular mapping of adult-plant race-specific leaf rust resistance gene *Lr12* in bread wheat. Mol. Breed. 28, 137–142. 10.1007/s11032-010-9467-4

[B83] Tehseen M. M. Tonk F. A. Tosun M. Randhawa H. S. Kurtulus E. Ozseven I. (2022). QTL mapping of adult plant resistance to stripe rust in a doubled haploid wheat population. Front. Genet. 13, 900558. 10.3389/fgene.2022.900558 35646084 PMC9131033

[B84] Wang H. Zou S. Li Y. Lin F. Tang D. (2020). An ankyrin-repeat and WRKY-domain-containing immune receptor confers stripe rust resistance in wheat. Nat. Commun. 11, 1353. 10.1038/s41467-020-15139-6 32170056 PMC7070047

[B85] Wang J. Li Z. Shi L. Zhu L. Ren Z. Li X. (2015). QTL mapping for adult-plant leaf rust resistance genes in Chinese wheat cultivar weimai 8. Czech J. Genet. Plant Breed. 51 (3), 79–85. 10.17221/51/2015-CJGPB

[B86] Wang J. Tao F. An F. Zou Y. Tian W. Chen X. (2016). Wheat transcription factor TaWRKY70 is positively involved in high-temperature seedling plant resistance to *Puccinia striiformis* f. sp. *tritici* . Mol. Plant Pathol. 10.1111/mpp.12425 PMC6638234 27145738

[B87] Wang J. K. Li H. H. Zhang L. Y. Meng L. (2012). “QTL IciMapping version 3.2,” in Qunatitative genetics group, inst. Of crop sci., Chinese acad. Of Agric.Sci. (Beijing. Available at: http://www.isbreeding.net (Accessed September 16, 2013).

[B88] Wang N. Tang C. Fan X. He M. Gan P. Zhang S. (2022). Inactivation of a wheat protein kinase gene confers broad-spectrum resistance to rust fungi. Cell 185 (16), 2961–2974. 10.1016/j.cell.2022.06.027 35839760

[B89] Wellings C. R. (2011). Global status of stripe rust: a review of historical and current threats. 179(1), 129–141. 10.1007/s10681-011-0360-y

[B90] Wu J. Gao J. Bi W. Zhao J. Yu X. Li Z. (2019). Genome-wide expression profiling of genes associated with the *Lr47*-mediated wheat resistance to leaf rust (*Puccinia triticina*). Int. J. Mol. Sci. 20 (18), 4498. 10.3390/ijms20184498 31514396 PMC6769777

[B91] Xu Y. Zou S. Zeng H. Wang W. Wang B. Wang H. (2022). NAC transcription factor TuNAC69 contributes to ANK-NLR-WRKY NLR-mediated stripe rust resistance in the diploid wheat *Triticum urartu* . Int. J. Mol. Sci. 23 (1), 564. 10.3390/ijms23010564 35008990 PMC8745140

[B92] Ye X. Li J. Cheng Y. Yao F. Long L. Yu C. (2019). Genome-wide association study of resistance to stripe rust (*Puccinia striiformis* f. sp. *tritici*) in Sichuan wheat. BMC Plant Biol. 19 (1), 147. 10.1186/s12870-019-1764-4 30991940 PMC6469213

[B93] Yessenbekova G. T. Kokhmetova A. M. Kampitova G. A. Radivoje J. (2016). Sources and donors for soft wheat selection by resistance to yellow rust. Biosci. Biotechnol. Res. Asia 13 (2), 693–700. 10.13005/bbra/2086

[B94] Yuan F.-P. Zeng Q.-D. Wu J.-H. Wang Q.-L. Yang Z.-J. Liang B.-P. (2018). QTL mapping and validation of adult plant resistance to stripe rust in Chinese wheat landrace humai 15. Front. Plant Sci. 9, 968. 10.3389/fpls.2018.00968 30026752 PMC6041984

[B95] Zhang C. Huang L. Zhang H. Hao Q. Lyu B. Wang M. (2019). An ancestral NB-LRR with duplicated 3′ UTRs confers stripe rust resistance in wheat and barley. Nat. Commun. 10 (1), 1–12. 10.1038/s41467-019-11872-9 31492844 PMC6731223

[B96] Zhang P. Lan C. Singh R. P. Huerta-Espino J. Li Z. Lagudah E. (2022). Identification and characterization of resistance loci to wheat leaf rust and stripe rust in Afghan landrace “KU3067”. Front. Plant Sci. 13, 894528. 10.3389/fpls.2022.894528 35837449 PMC9274257

[B97] Zhang P. Li X. Gebrewahid T. W. Liu H. Xia X. He Z. (2019). QTL mapping of adult-plant resistance to leaf and stripe rust in wheat cross SW 8588/thatcher using the wheat 55K SNP array. Plant Dis. 103 (12), 3041–3049. 10.1094/PDIS-02-19-0380-RE 31613193

[B98] Zhang P. Yin G. Zhou Y. Qi A. Gao F. Xia X. (2017). QTL mapping of adult-plant resistance to leaf rust in the wheat cross zhou 8425B/Chinese spring using high-density SNP markers. Front. Plant Sci. 8, 793. 10.3389/fpls.2017.00793 28559910 PMC5432574

[B99] Zhang Y. Geng H. Cui Z. Wang H. Liu D. (2021). Functional analysis of wheat NAC transcription factor, TaNAC069, in regulating resistance of wheat to leaf rust fungus. Front. Plant Sci. 12, 604797. 10.3389/fpls.2021.604797 33790919 PMC8005738

[B100] Zhu Z. Cao Q. Han D. Wu J. Wu L. Tong J. (2023). Molecular characterization and validation of adult-plant stripe rust resistance gene *Yr86* in Chinese wheat cultivar Zhongmai 895. Theor. Appl. Genet. 136 (6), 142. 37247049 10.1007/s00122-023-04374-2

[B101] Ziyaev Z. M. Sharma R. C. Nazari K. Morgounov A. I. Amanov A. A. Ziyadullaev Z. F. (2011). Improving wheat stripe rust resistance in Central Asia and the Caucasus. Euphytica 179, 197–207. 10.1007/s10681-010-0305-x

