# Supplementary material for: QTL mapping for seedling and adult plant resistance to stripe and leaf rust in two winter wheat populations
Source: Front Genet. 2023 Nov 23;14:1265859. doi: 10.3389/fgene.2023.1265859 (PMC10702562; doi:10.3389/fgene.2023.1265859)
Supplement: Supplementary file 1 [file DataSheet2.PDF]

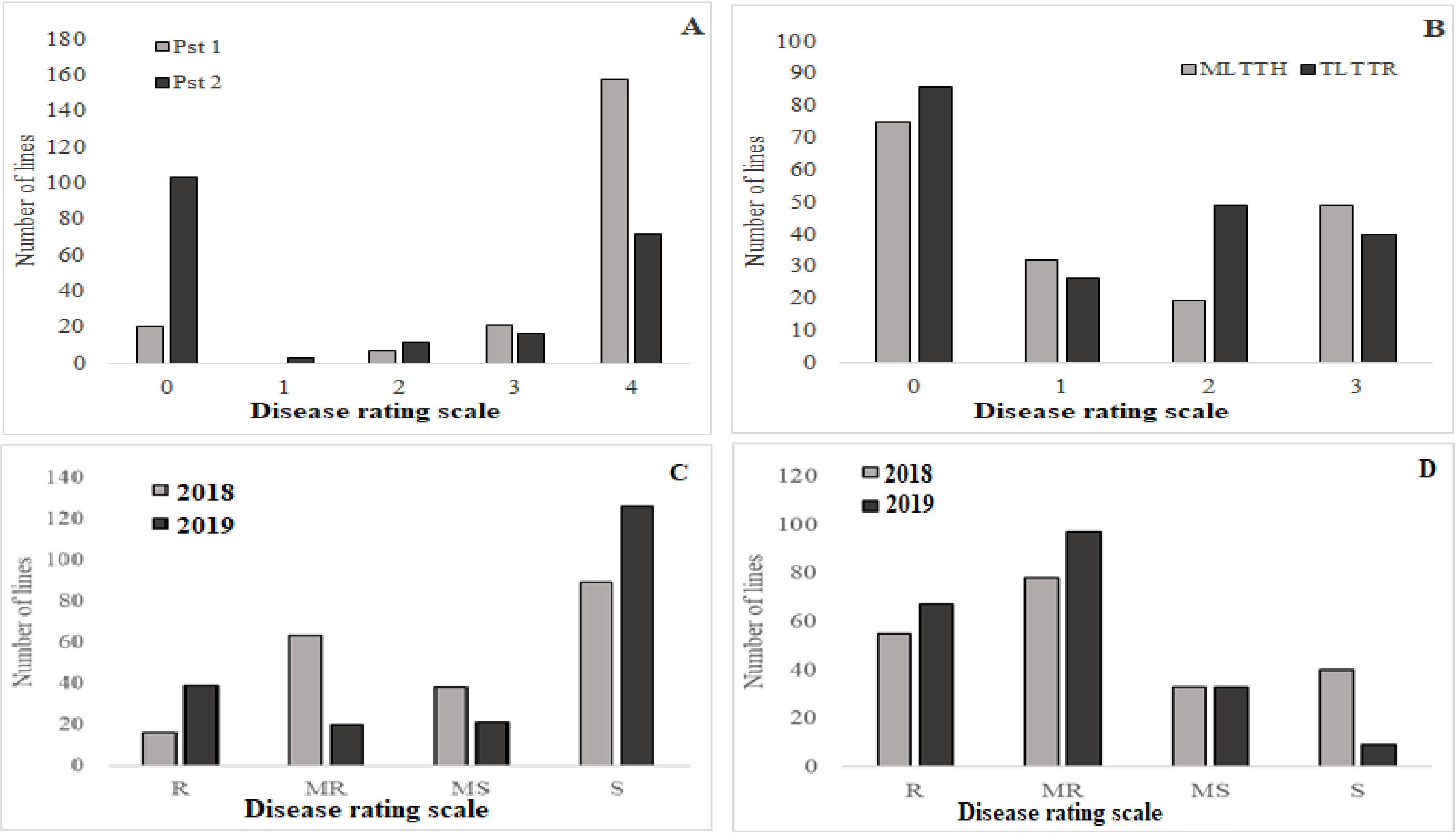

Supplementary Figure 2. Frequency distribution of Almaly x Avocet (S) recombinant inbred lines (RILs) for stripe rust (A, seedling and C, field) and leaf rust (B, seedling and D, field) seedling infection type and adult plant resistance in different disease score groups
